# Evolution of a Strategy for the Total Synthesis of the *Ganoderma* Meroterpenoid Ganoapplanin

**DOI:** 10.1002/ceur.202500020

**Published:** 2025-04-26

**Authors:** Nicolas Müller, Ondřej Kováč, Alexander Rode, Daniel Atzl, Clemens Dietrich, Ana V. Serna, Sebastian Schaar, Antonio Paparesta, Julian Lichtenegger, Thomas Magauer

**Affiliations:** Department of Organic Chemistry and Center for Molecular Biosciences, University of Innsbruck, Innrain 80–82, 6020 Innsbruck, Austria; Department of Organic Chemistry and Center for Molecular Biosciences University of Innsbruck, Innrain 80–82, 6020 Innsbruck, Austria; Department of Organic Chemistry, Palacký University Olomouc, tř. 17. listopadu 1192/12, 77900 Olomouc, Czech Republic; Department of Organic Chemistry and Center for Molecular Biosciences University of Innsbruck, Innrain 80–82, 6020 Innsbruck, Austria; Department of Organic Chemistry and Center for Molecular Biosciences University of Innsbruck, Innrain 80–82, 6020 Innsbruck, Austria; Department of Chemistry, University of Pennsylvania, 231 South 34th Street, Philadelphia, PA 19104, USA; Department of Organic Chemistry and Center for Molecular Biosciences University of Innsbruck, Innrain 80–82, 6020 Innsbruck, Austria

**Keywords:** *Ganoderma*, meroterpenoids, natural products, radicals, total synthesis

## Abstract

Herein, a detailed account of the efforts leading to the recently published synthesis of the *Ganoderma* meroterpenoid ganoapplanin, a natural product identified as an inhibitor of T-type voltage-gated calcium channels, is provided. Ganoapplanin, which was isolated as a racemate from the fungus *Ganoderma applanatum* in 2016, features a complex structure, including a characteristic spiro bisacetal structure, a highly functionalized tetra-*ortho*-substituted biaryl motif, and a propellane-like dioxatricyclo[4.3.3.0]dodecane scaffold. While the southern terpenoid fragment is available via a diastereoselective titanium-mediated iodolactonization, considerable efforts are required to fuse this fragment with various aromatic fragments. The breakthrough was achieved by a highly efficient two-component coupling strategy that simultaneously fuses the fragments and establishes the crucial biaryl bond. This transformation involves an intramolecular 6-*exo*-trig radical addition of a quinone monoacetal, followed by an intermolecular aldol addition. Finally, strategic late-stage oxidations enabled the formation of the characteristic spiro bisacetal motif and the completion of the synthesis of ganoapplanin.

## 1 Introduction

**Figure 1:**
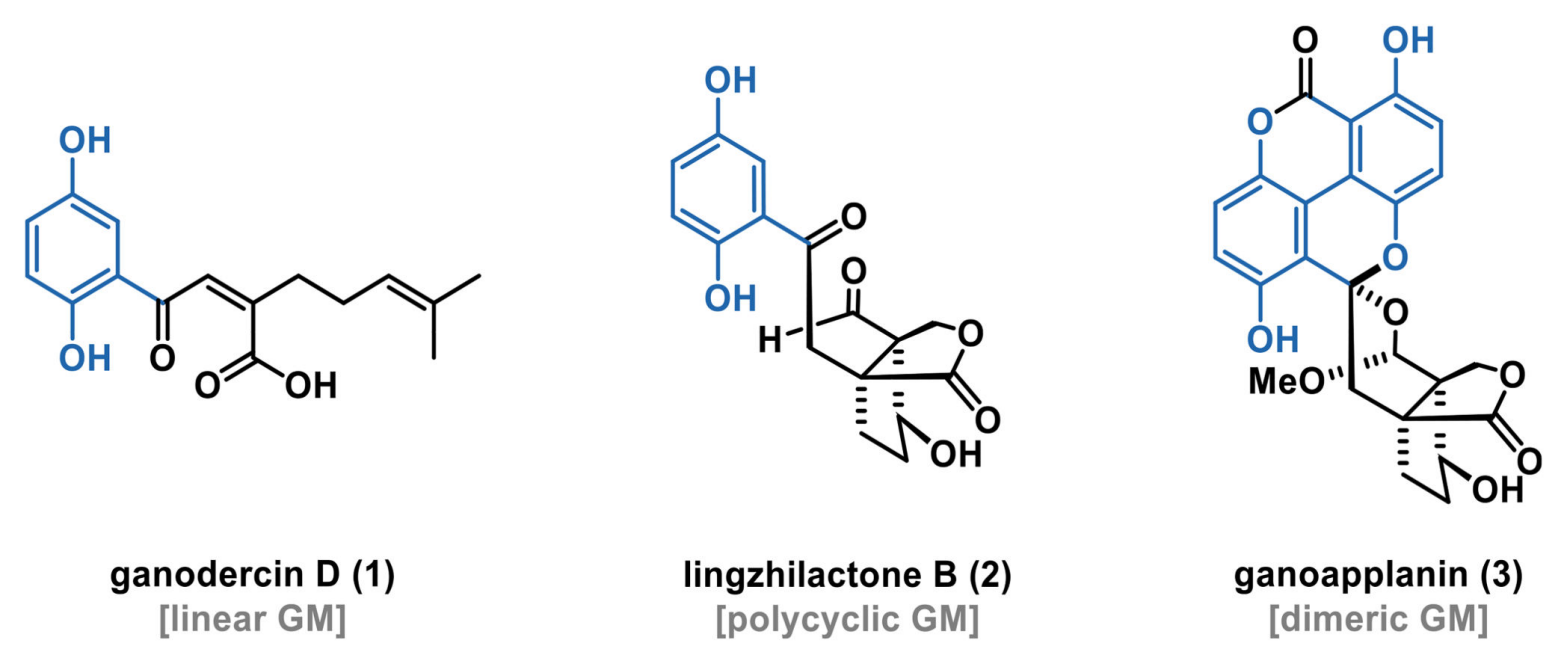
Selected structures of GMs and their classification.

*Ganoderma*, a genus of wood decay fungi frequently used in traditional Chinese medicine, has been a subject of intense research due to its diverse array of bioactive compounds.^[1]^ Among the various classes of compounds isolated from *Ganoderma* species, meroterpenoids are particularly noteworthy for their structural complexity and significant bioactivity profiles including antioxidant, antifibrotic, and antimicrobial activities. *Ganoderma* meroterpenoids (GMs) can be classified based on their structural complexity into three distinct subclasses: linear, polycyclic, and dimeric GMs (Figure 1).^[1]^

Linear GMs are characterized by a hydroquinone group linked to a linear terpenoid chain, as exemplified by ganodercin D (**1**).^[2]^ In contrast, polycyclic GMs, such as lingzhilactone B (**2**),^[3]^ feature a hydroquinone motif connected to a polycyclic ring system. Dimeric GMs, including ganoapplanin (**3**),^[4]^ consist of two hydroquinone units attached to a terpenoid backbone, resulting in greater structural complexity.

From a biosynthetic perspective, GMs are constructed from 4-hydroxybenzoic acid (**4**) and GPP (geranyl diphosphate, **5**), with the former originating from the shikimic acid pathway. In the first step, **4** is attached to GPP by a geranyltransferase, followed by oxidative decarboxylation to yield the hydroquinone intermediate **6**.^[5]^ This compound can then undergo allylic oxidation to form linear GMs, such as ganodercin D (**1**). In addition, oxidative processes can trigger cyclization reactions leading to a wide range of structurally diverse polycyclic meroterpenoids.

**Scheme 1:**
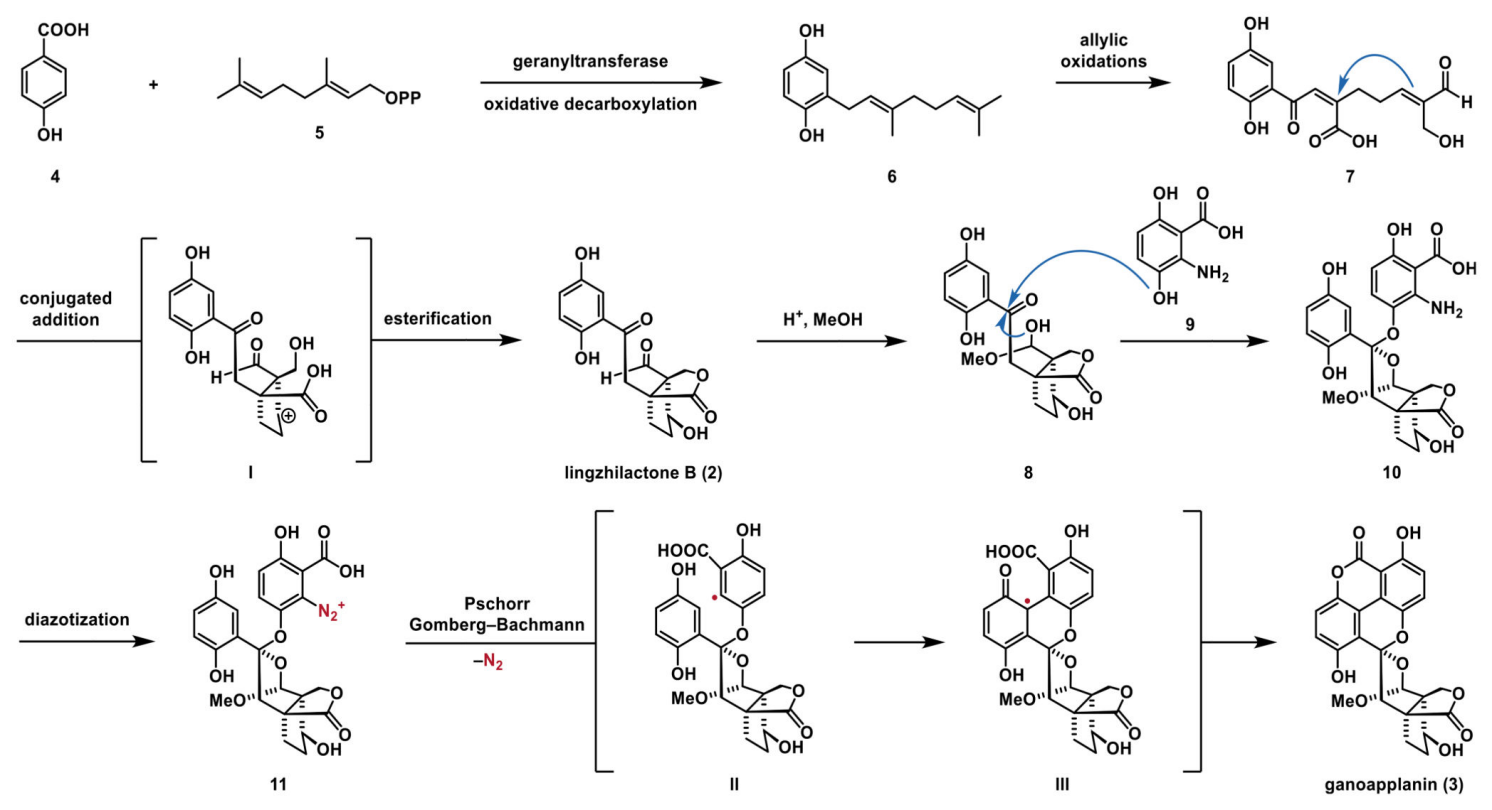
Proposed biosynthetic pathway to lingzhilactone B (**2**) and ganoapplanin (**3**).

For instance, lingzhilactone B (**3**) is thought to form via the oxidation of intermediate **6** to precursor **7**, which then undergoes an intramolecular conjugate addition to generate cyclopentyl carbocation **I**. Further transformations, including esterification and interception from water, lead to the bicyclic structure of lingzhilactone B (**2**).^[5]^ In the later stages of the proposed biosynthesis of ganoapplanin (**3**), methanol and anthranilic acid **9** react with lingzhilactone B (**2**) to form acetal **10**. To construct the aryl–aryl bond, a diazotization of the aniline group followed by a Pschorr–Gomberg–Bachmann radical cyclization was proposed.^[6,7]^ The biosynthesis is completed through aromatization and lactonization, culminating in the formation of ganoapplanin (**3**) (Scheme 1).^[4]^

The unique structural features and compelling biological activities of GMs make them highly appealing targets for total synthesis.^[8]^ Polycyclic GMs, such as cochlearol B^[9–12]^ and lingzhiol,^[13–19]^ have been synthesized multiple times. Recently, we also reported the total synthesis of lingzhilactone B (**2**), meroapplanin B, lingzhiol, and other related polycyclic GMs.^[19]^ The synthesis of dimeric GMs remains relatively underexplored, with only a few examples reported, such as cochlearoid B.^[20]^

**Scheme 2:**
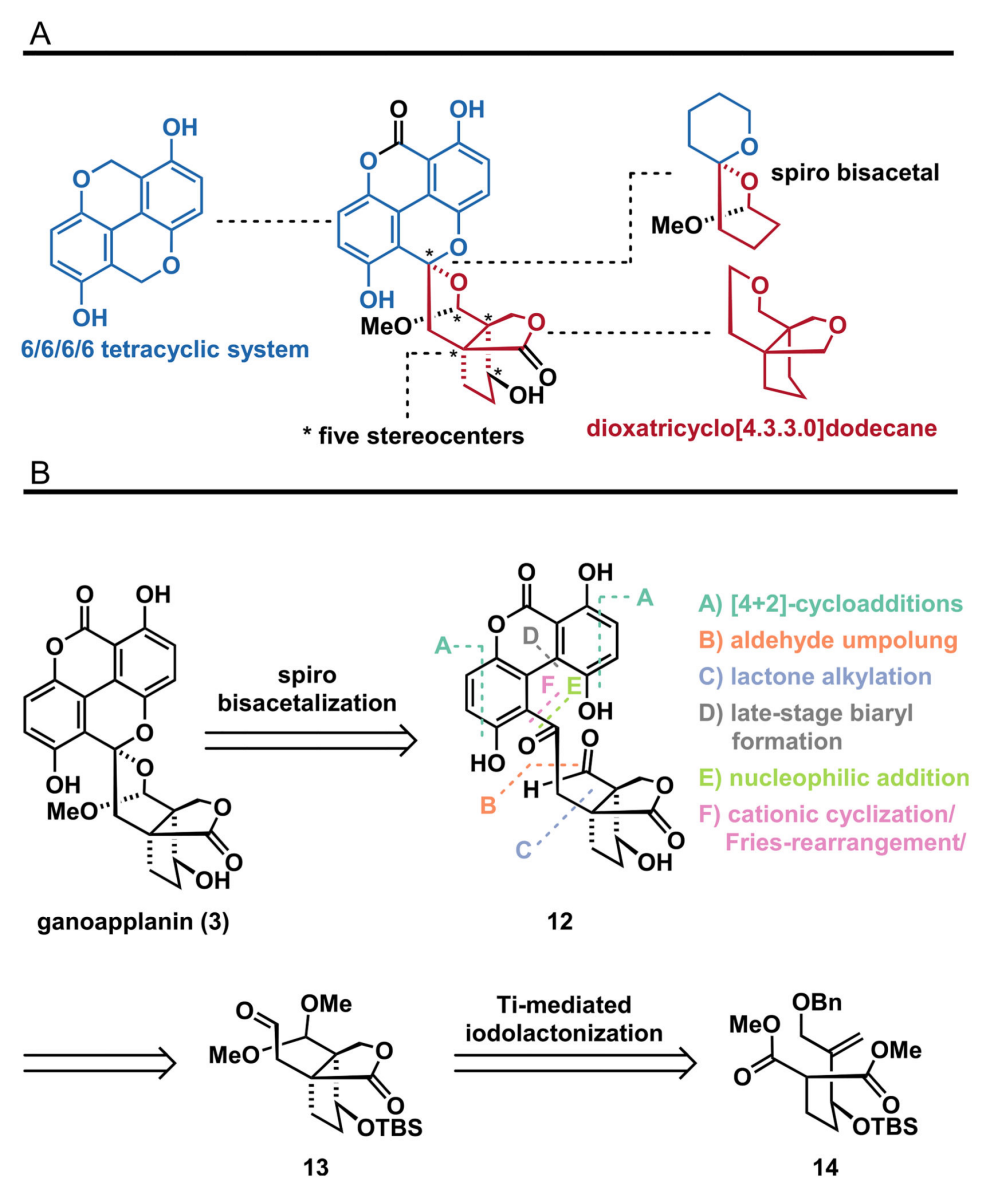
Structural features of ganoapplanin (**3**) and attempted key-steps: A) structural features and B) key bond disconnections.

Ganoapplanin (**3**), first isolated in racemic form by Qiu from *Ganoderma applanatum* in 2016,^[4]^ is particularly remarkable from a structural standpoint. It contains five contiguous stereocenters, including two quaternary centers, and features a unique spiro bisacetal framework. This intricate structure is composed of a 6/6/6/6 tetracyclic system, incorporating a tetra-*ortho*-substituted biaryl motif and a dioxatricyclo[4.3.3.0]dodecane propellane-like scaffold (Scheme 2A). Given the traditional use of *Ganoderma* fungi extracts in Chinese medicine as an adjuvant for central nervous system disorders,^[21]^ the neuroprotective potential of ganoapplanin (**3**) was also investigated. In the course of this study, inhibition of T-type voltage-gated calcium channels was revealed for a racemic mixture of **3** (IC_50_ of 36.6 μM).^[4]^ Based on these results, ganoapplanin (**3**) shows potential as a lead compound for the development of novel therapeutics against neurodegenerative diseases such as epilepsy or Parkinson’s disease.^[22,23]^ In recent years, our group has developed various synthetic methods for constructing highly functionalized arenes^[24]^ and heteroarenes.^[25]^ We have also successfully completed the total syntheses of natural products featuring similar polysubstituted aromatic frameworks.^[24,26,27]^ As these methods were unsuitable for the unique structure of ganoapplanin (**3**), in particular its highly congested central region, including the tetra-*ortho*-substituted biaryl motif, we set out to develop new synthetic strategies.

Herein, we report a full account of these attempts and present the development of a synthetic strategy that enabled us to accomplish the first total synthesis of ganoapplanin (**3**).^[28]^

## 2 Results and Discussion

### 2.1 Retrosynthetic Analysis

Considering the potential instability of the spiro bisacetal moiety, we opted for its late-stage construction and therefore traced back ganoapplanin (**3**) to hydroquinone **12** (Scheme 2B). To build up the latter, we designed several key steps, relying on (**A**) [4 + 2]-cycloadditions, (**B**) umpolung reactions of aldehydes, (**C**) alkylations of lactones, (**D**) late-stage biaryl formations, (**E**) nucleophilic additions, and (**F**) Fries rearrangements/cationic cyclizations. The precursors for these key transformations can be traced back to lactone **13**, which resembles the southern fragment of ganoapplanin (**3**). Inspired by seminal work of Taguchi,^[29,30]^ we envisioned the construction of this lactone by a titanium(IV)-mediated iodolactonization of alkene **14**.

### 2.2 Synthesis of the Southern Fragment of Ganoapplanin

**Scheme 3:**
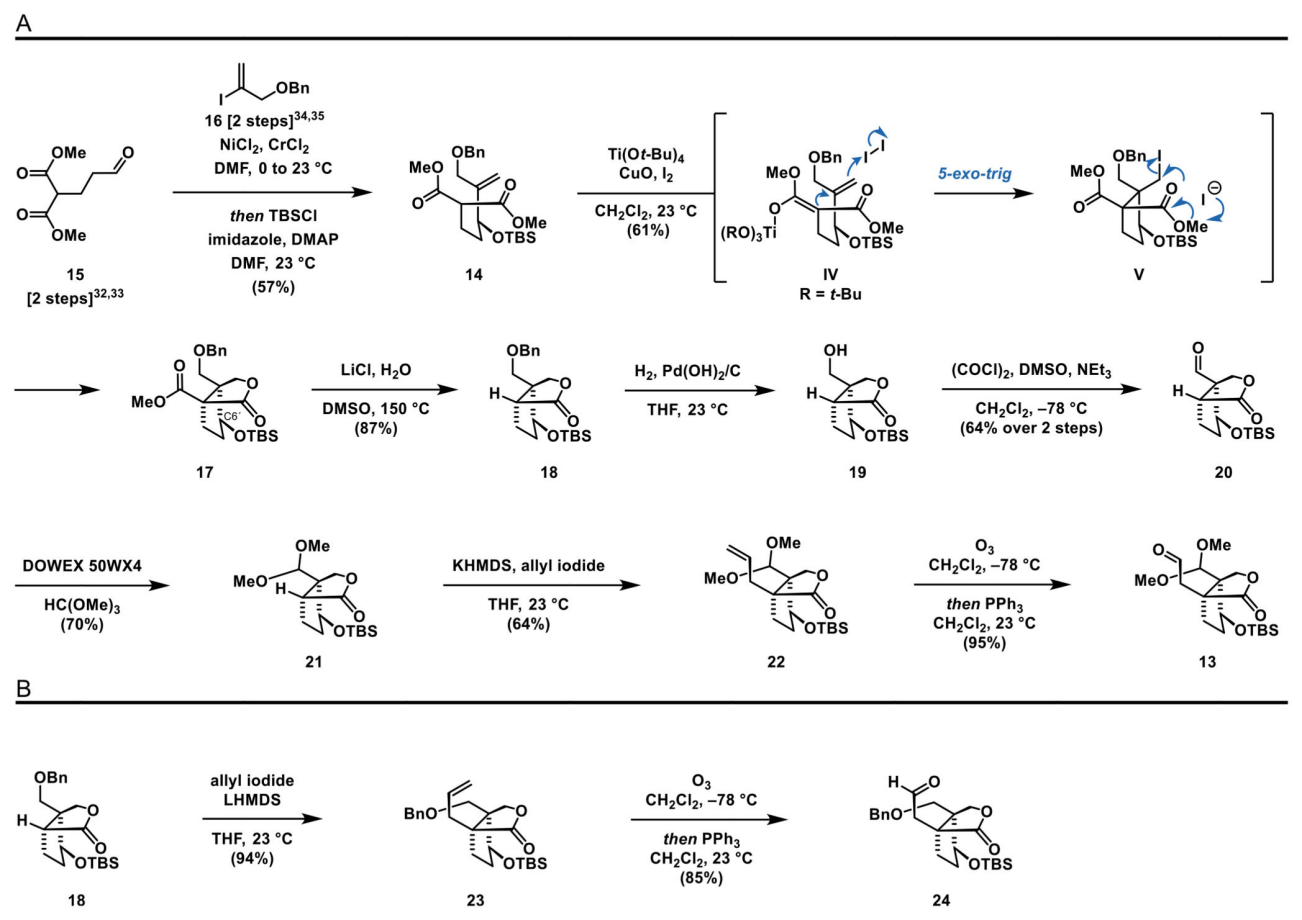
Syntheses of the southern fragments **13** and **24**: A) synthesis of aldehyde **13** and B) synthesis of aldehyde **24**.

We commenced our synthetic endeavors toward ganoapplanin (**3**) with a Nozaki–Hiyama–Kishi reaction^[31]^ between aldehyde **15**^[32,33]^ and vinyl iodide **16**^[34,35]^ followed by in situ TBS protection of the formed secondary alcohol gave access to silyl ether **14** (Scheme 3A). To forge the bicyclic lactone structure, we relied on an iodocarbocyclization reaction, using titanium(IV) *tert*-butoxide, copper(ll) oxide, and iodine.^[29,30]^ Under these conditions, **14** underwent a 5-*exo*-trig cyclization followed by lactonization, resulting in the formation of the bicyclic lactone **17** as a single diastereomer on a decagram scale in 61% yield. The reaction effectively established two quaternary centers during the 5-*exo*-trig cyclization (**IV** to **V**), achieving the desired relative configuration at C6′. Moving forward, we carried out a Krapcho decarboxylation (LiCl, H_2_O, DMSO, 140 °C) to remove the methyl ester of **17**.^[36]^ An attempt to perform the debenzylation of **18** with palladium on charcoal under a hydrogen atmosphere resulted in low reactivity. However, upon increasing the pressure to 40 bar and using Pearlman’s catalyst, we were able to realize the desired transformation. The resulting intermediate **19** was then subjected to a Swern oxidation, yielding aldehyde **20** in 64% over two steps.^[37,38]^ With aldehyde **20** in hand, we proceeded to attempt the protection of the carbonyl function as a dimethyl acetal. Surprisingly, this step proved more difficult than expected, as commonly used methods involving Brønsted or Lewis acids in the presence of methanol led either to decomposition or failed to initiate the reaction (see the Supporting Information for details). To our satisfaction, using the acidic resin Dowex 50WX4 in combination with trimethyl orthoformate yielded the desired dimethylacetal **21** in 70% yield.^[39]^

The installation of the adjacent quaternary stereocenter was completed by treating the lactone **21** with potassium bis(trimethylsilyl)amide (KHMDS) and allyl iodide at 23 °C, yielding alkene **22** in 64% yield. The final step toward aldehyde **13** involved the oxidative cleavage of alkene **22** (O_3_, *then* PPh_3_), which provided aldehyde **13** in 95% yield. Similarly, we were able to access alkene **23** and aldehyde **24** via direct allylation of lactone **18**, followed by ozonolysis (Scheme 3B).

#### 2.2.1 Construction of the Biaryl Moiety via [4 + 2]-Cycloadditions

**Scheme 4:**
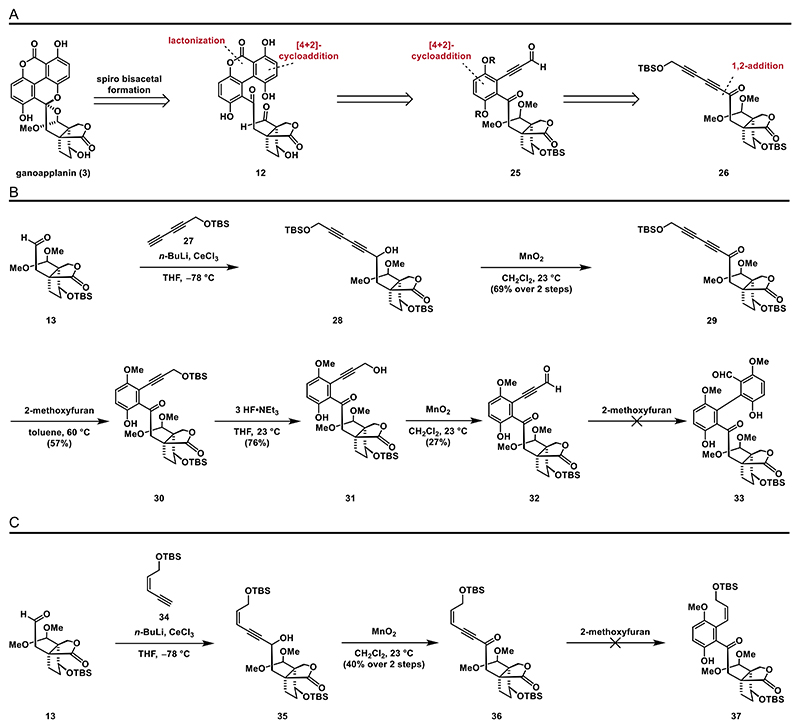
[4 + 2]-Cycloaddition approaches: A) retrosynthetic analysis for strategy **A**; B) [4 + 2]-cycloaddition employing a diyne, and C) [4 + 2]-cycloaddition employing an enyne.

After establishing a synthetic route to aldehyde **13**, we were eager to explore our first proposed strategy for the synthesis of the biaryl moiety of ganoapplanin (**3**), which involved a couple of [4 + 2]-cycloadditions of a diyne moiety with 2-methoxyfuran (Scheme 4A).^[40–43]^

We commenced this synthetic approach with the preparation of diyne **27** (see the Supporting Information for details). Upon deprotonation using *n*-BuLi, the corresponding lithium acetylide was formed, which was then transmetalated with cerium(lll) tri-chloride, forming a suitable nucleophile to attack aldehyde **13** to give secondary alcohol **28** as an inconsequential diastereomeric mixture (1.4:1). Oxidation with manganese(IV) oxide gave access to ketone **29** and set the stage for the first [4 + 2]-cycloaddition. Treating acetylenic ketone **29** with 2-methoxyfuran allowed us to obtain hydroquinone **30** in good yields (57%). In order to activate the remaining aldehyde moiety for the upcoming second [4 + 2]-cycloaddition, we decided to install an electron-withdrawing group on the terminal alkyne position. To this end, desilylation using 3 HF·NEt_3_ provided propargylic alcohol **31** in 76% yield. Even though the oxidation of **31** with MnO_2_ was successful, the desired aldehyde **32** turned out to be unstable and was, therefore, isolated in only low yield (27%). Unfortunately, the key [4 + 2]-cycloaddition of **32** with 2-methoxyfuran proved to be fruitless under several screened conditions, including treatment with excess diene at elevated temperatures (60 °C) and/or applying high pressure (14 kbar)^[44]^ as decomposition of the starting material was observed (Scheme 4B).

Surprisingly, an attempt to realize an analogous [4 + 2]-cycloaddition of 2-methoxyfuran and enyne **36**, accessible via a comparable 1,2-addition/oxidation sequence, resulted in no reaction despite the similarity of the dienophiles **29** and **36**.

#### 2.2.2 Aldehyde Umpolung

**Scheme 5:**
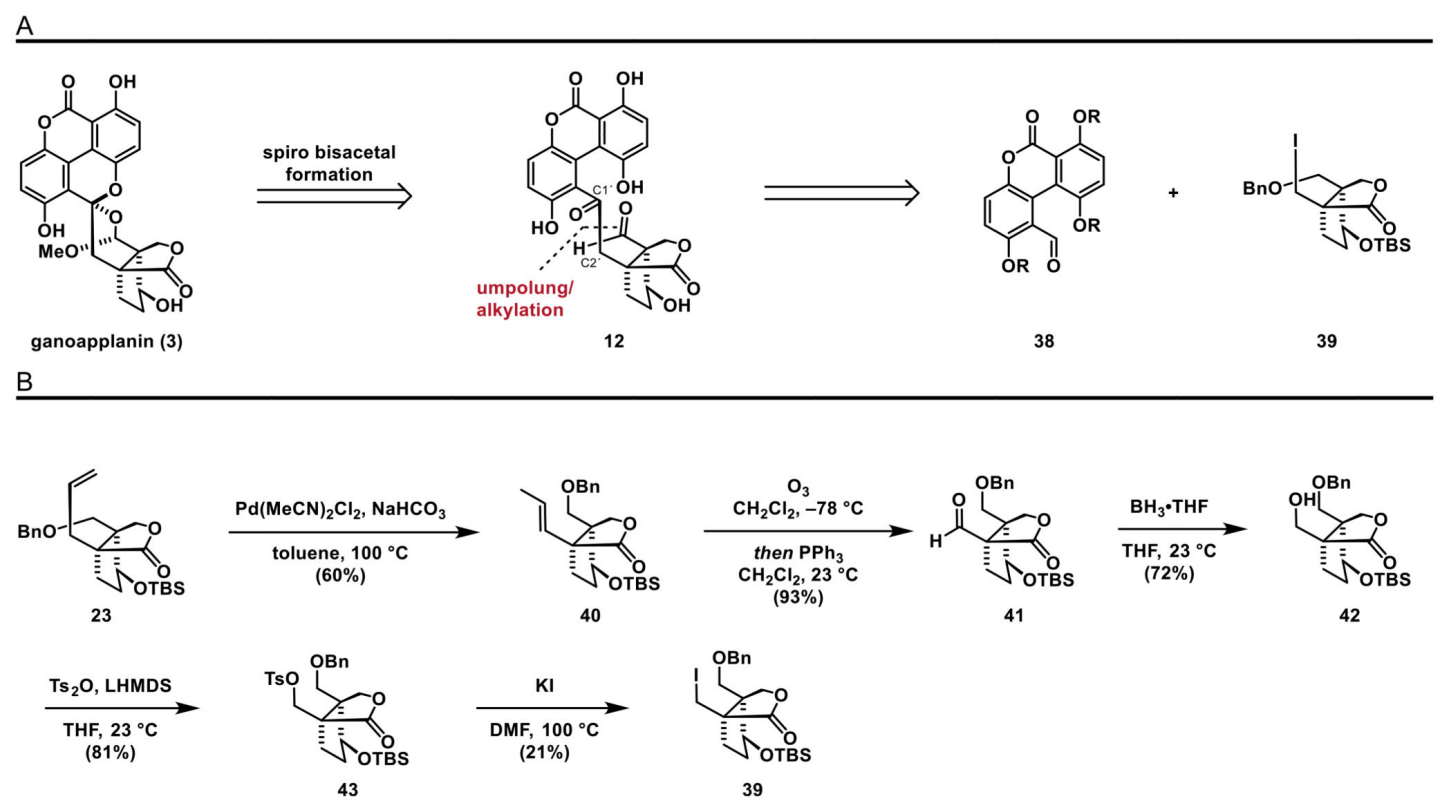
Retrosynthetic analysis of the umpolung/alkylation approach and synthesis of the southern component **39**: A) retrosynthetic analysis for strategy **B** and B) synthesis of alkyl **39**.

As the construction of the biaryl motif of ganoapplanin (**3**) turned out to be more difficult than expected, we considered an alternative approach, which is based on the synthesis of an aromatic fragment, substituted with an aldehyde moiety, via cross-coupling reactions. Subsequent umpolung of aldehyde **38**,^[45]^ followed by alkylation with alkyl iodide **39**, should forge the C2-linker (C−C bond between C1′ and C2′) between both fragments (Scheme 5A). To investigate this strategy, we commenced with the synthesis of alkyl iodide **39**, which was conducted in five steps starting from lactone **23** (Scheme 5B). The process began with an isomerization of the terminal olefin of **23** to internal olefin **40** using Pd(MeCN)_2_Cl_2_ in toluene at 100 °C.^[46]^ Ozonolysis of the double bond yielded aldehyde **41**, which was then reduced to the corresponding alcohol **42** using the borane–tetrahydrofuran complex. The desired alkyl iodide **39** was accessed upon tosylation and iodination with potassium iodide. Having achieved the synthesis of alkyl iodide **39**, we turned our focus to the synthesis of the aromatic fragment. Thus, we designed tricycles **49** and **50** with the desired substitution pattern including a protected benzylic alcohol, which could then be further elaborated to the corresponding benzaldehyde **38** required for the umpolung/alkylation approach.

**Scheme 6:**
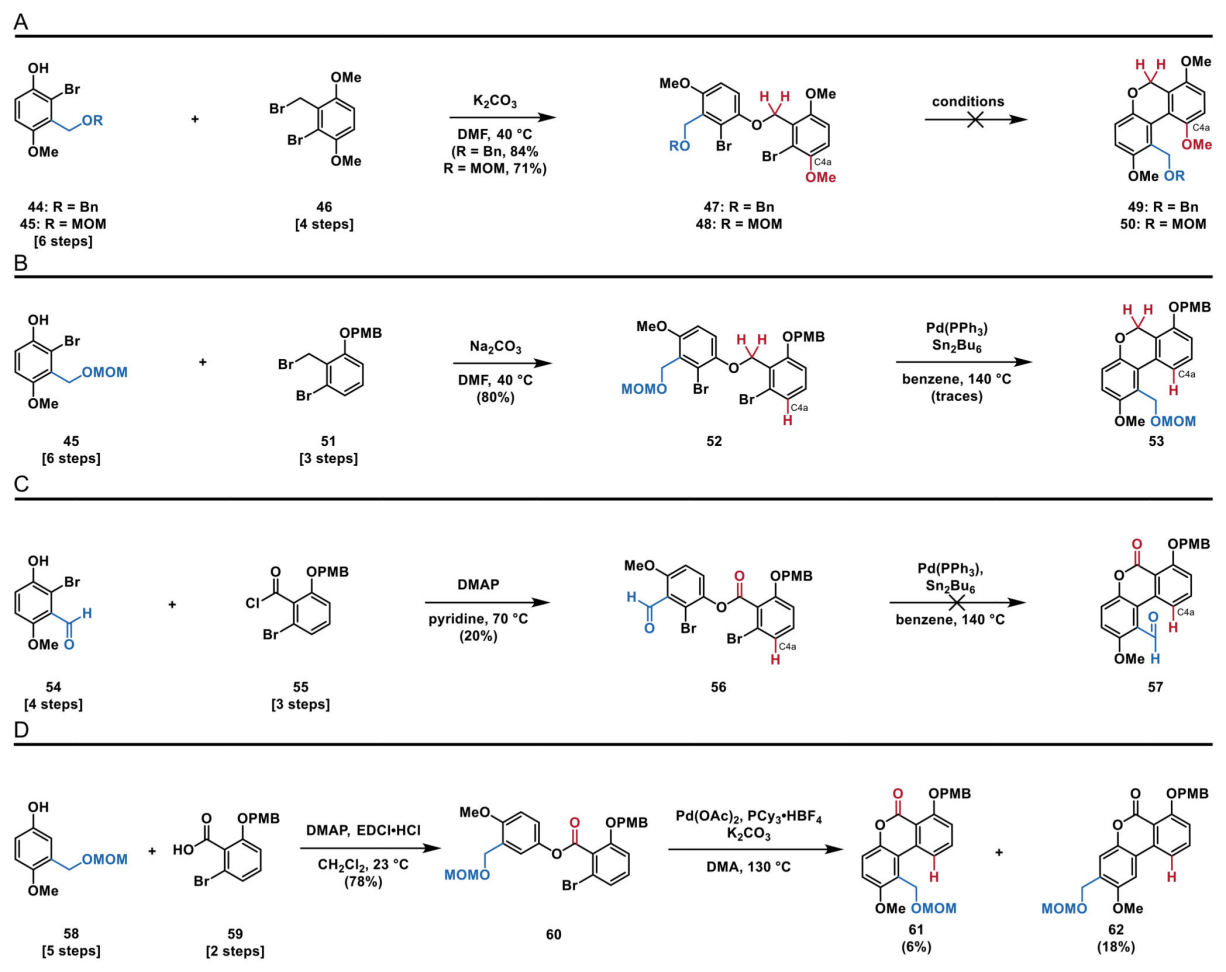
Attempted intramolecular coupling reactions: A) attempted intramolecular coupling of ethers **47** and **48**; B) Stille–Kelly coupling of ether **52**; C) Stille–Kelly coupling of ester **56**; and D) C–H functionalization of ester **60**.

We initiated this campaign with the synthesis of phenols **44** and **45** and treated them with benzyl bromide **46** under basic conditions to access the bis-halogenated ethers **47** and **48** (see the Supporting Information for details). To construct the crucial aryl–aryl bond, we investigated several metal catalyzed cross-coupling reactions. Unfortunately, these attempts exclusively resulted in decomposition of **47** and **48** and were unsuitable to form either **49** or **50** (Scheme 6A). We reasoned that the systems we used were challenging substrates for cross-coupling reactions for two reasons: first, due to the steric hindrance caused by a total of four *ortho* substituents next to the bromine substituents, and second, due to the electron richness caused by several electron donating substituents.

To overcome the issue regarding sterical hindrance, we decided to synthesize bis-halogenated ether **52** (see the Supporting Information for details), which is lacking the phenol group at C4a, from phenol **45** and benzyl bromide **51**. Unfortunately, attempts to form the aryl–aryl bond, using Stille–Kelly cross-coupling conditions (Pd(PPh_3_)_4_, Sn_2_Bu_6_, 140 °C), only gave trace amounts of biaryl **53** (Scheme 6B).^[26,47,48]^

We then set out to reduce electron richness of the system and synthesized ester **56** (see the Supporting Information for details) from phenol **54** and acyl chloride **55**. Surprisingly, when subjecting **56** to the same conditions, only decomposition was observed, presumably due to instability of the aldehyde moiety (Scheme 6C). A C–H functionalization approach involving the treatment of ester **60** (see the Supporting Information for details) with Pd(OAc)_2_, PCy_3_·HBF_4_ and potassium carbonate at elevated temperatures (130 °C) in DMA^[49]^ led to a regioisomeric mixture of biaryl **62** and **61** in 3:1 ratio favoring the undesired sterically less hindered regioisomer (Scheme 6D). From these results, we concluded that ester **63** might be a suitable substrate, as it features the electron-withdrawing ester moiety, a sturdy MOM-protected benzylic alcohol and two bromo substituents, to avoid the formation of regioisomers. We therefore synthesized ester **63** by treating phenol **45** and acyl chloride **55** with DMAP in pyridine in almost quantitative yields (97%).

**Scheme 7:**
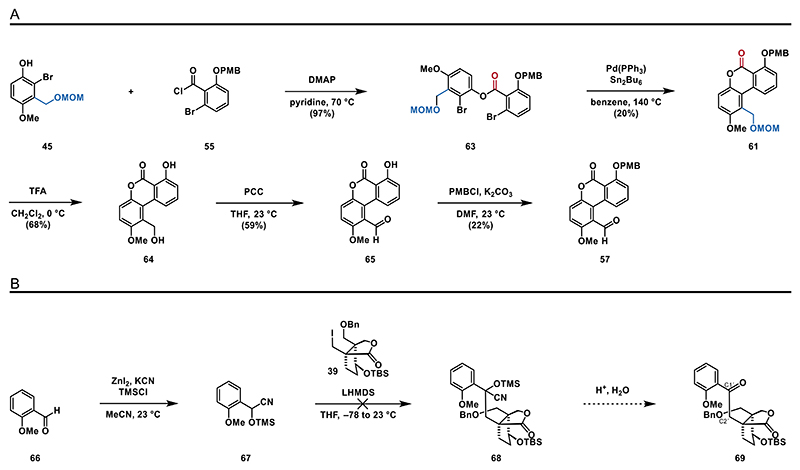
Synthesis of benzaldehyde **57** and umpolung/alkylation attempt: A) Stille–Kelly coupling of ester **63** and B) attempted umpolung/alkylation sequence.

Having this substrate in hand, we screened several Stille–Kelly cross-coupling conditions (see the Supporting Information for details) and found that treatment of **63** with Pd(PPh_3_)_4_ with Sn_2_Bu_6_ in benzene at elevated temperatures (140 °C) led to the formation of biaryl **61** in around 20% yield (Scheme 7A).

Moving on with protected biaryl **61**, we attempted the chemoselective deprotection of the MOM-ether; however, the PMB proved to be more labile. Therefore, we opted for a global deprotection with trifluoroacetic acid (TFA) to give benzylic alcohol **64**, which was then oxidized by treatment with pyridinium chlorochromate (PCC) to benzaldehyde **65**. Subsequently, the PMB protecting group was reinstalled to give **57**. As the construction of benzaldehyde **57** proved to be more challenging and inefficient than anticipated, the synthetic sequence yielded only small amounts of the target compound. Consequently, we decided to explore the key umpolung/alkylation transformation using a model substrate instead. Therefore, we synthesized cyanohydrin **67**^[50]^ from commercially available *ortho*-anisaldehyde (**66**). Attempts to deprotonate **67** with lithium bis(trimethylsilyl)amide (LHMDS) followed by exposure to alkyl iodide **39** at various temperatures failed to produce either **68** or ketone **69**. We hypothesized that the desired reaction was prevented by the steric hindrance of the neopentylic alkyl iodide (Scheme 7B).

#### 2.2.3 Lactone Alkylation

**Scheme 8:**
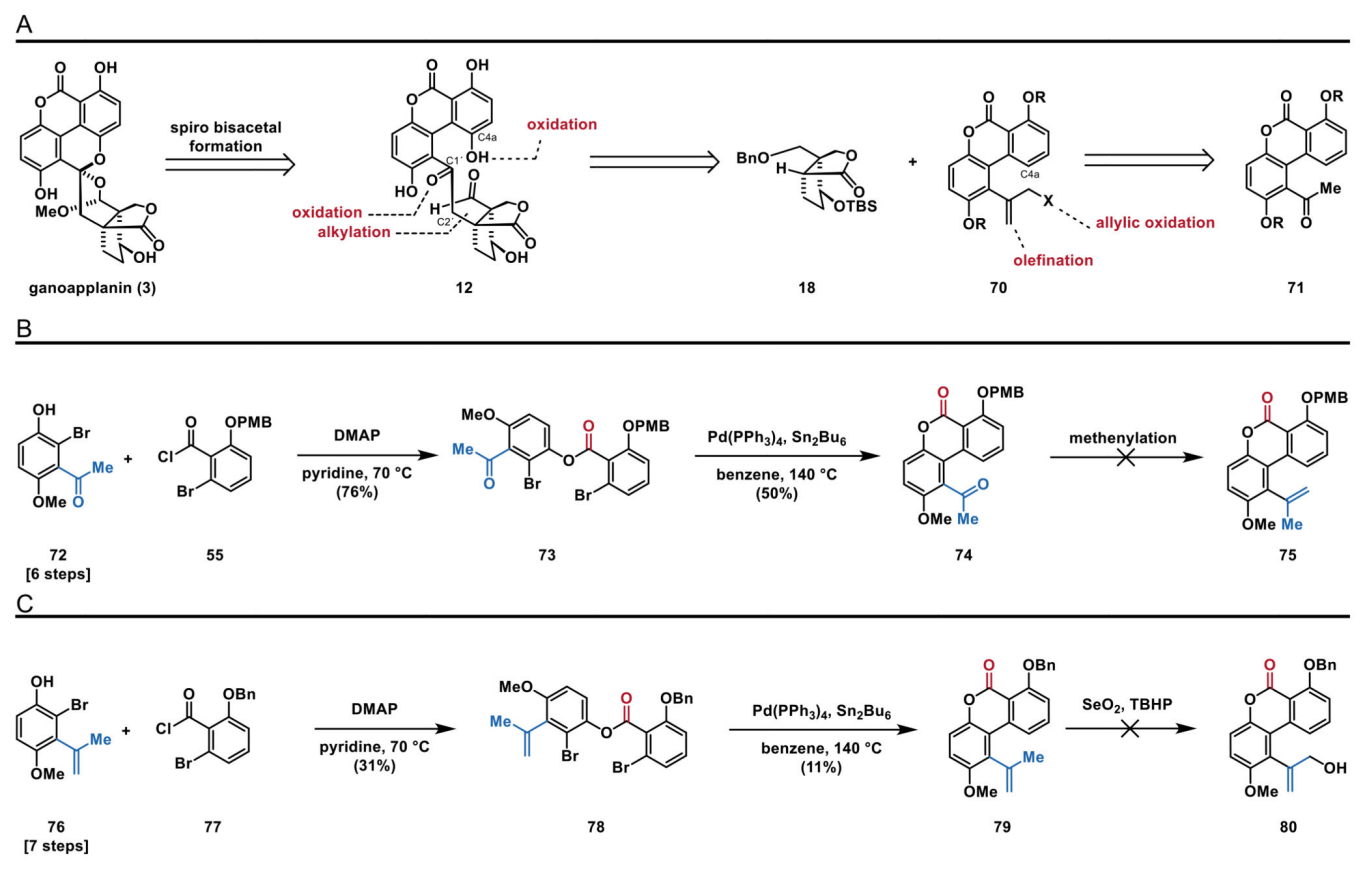
Lactone alkylation strategy: A) retrosynthetic analysis for strategy **C**; B) attempted formation of styrene **75**; and C) synthesis of styrene **79** and attempted functionalization of the allylic position.

Since our attempt to construct the C1′−C2′ bond between the aromatic motif and terpenoid fragment via an umpolung strategy was unsuccessful, we decided to discontinue this approach. Instead, we moved on to investigate the alkylation of lactone **18**. This approach builds on our prior success with allylating lactone **18** using allyl iodide and LHMDS. We reasoned that aromatic fragment **70**, substituted with a leaving group in the allylic position, should serve as a suitable electrophile for addition to the enolate derived from lactone **18**. This strategy would yield ketone **12** through oxidative cleavage of the olefin and oxidation at C4a to form the hydroquinone motif. **70** can be traced back to ketone **71** via olefination and functionalization of the allylic position (Scheme 8A).

To access ester **73** (see the Supporting Information for details), we performed an esterification of phenol **72** with acyl chloride **55** in the presence of 4-dimethylaminopyridine and pyridine. With **73** in hand, we were poised for another Stille–Kelly cross-coupling reaction. To our delight, the reaction conditions previously employed for synthesizing biaryl **61** also proved effective for the preparation of biaryl **74**. Notably, replacing the electron-donating MOM-protected alcohol (as in **63**) with an electron-withdrawing ketone group (as in **73**) increased the yield from 20 to 50%. Moving forward, we explored various conditions for the methenylation of ketone **74**. However, all olefination attempts (e.g., Wittig, Nysted)^[51]^ were unsuccessful, and we consistently reisolated the starting material. We suspect that steric hindrance around the ketone moiety may prevent the desired transformation (Scheme 8B).

To address the challenges with the olefination, we synthesized ester **78** (see the Supporting Information for details), which already carries the propene moiety. By applying our optimized Stille–Kelly cross-coupling conditions to this substrate, we were able to obtain the biaryl **79** in 11% yield. We attributed the low yield to the more electron-rich nature of the styrene moiety compared to the previously used ketone. With a limited supply of **79**, we attempted an allylic oxidation under modified Riley conditions;^[52,53]^ however, only decomposition of biaryl **79** was observed (Scheme 8C).

**Scheme 9:**
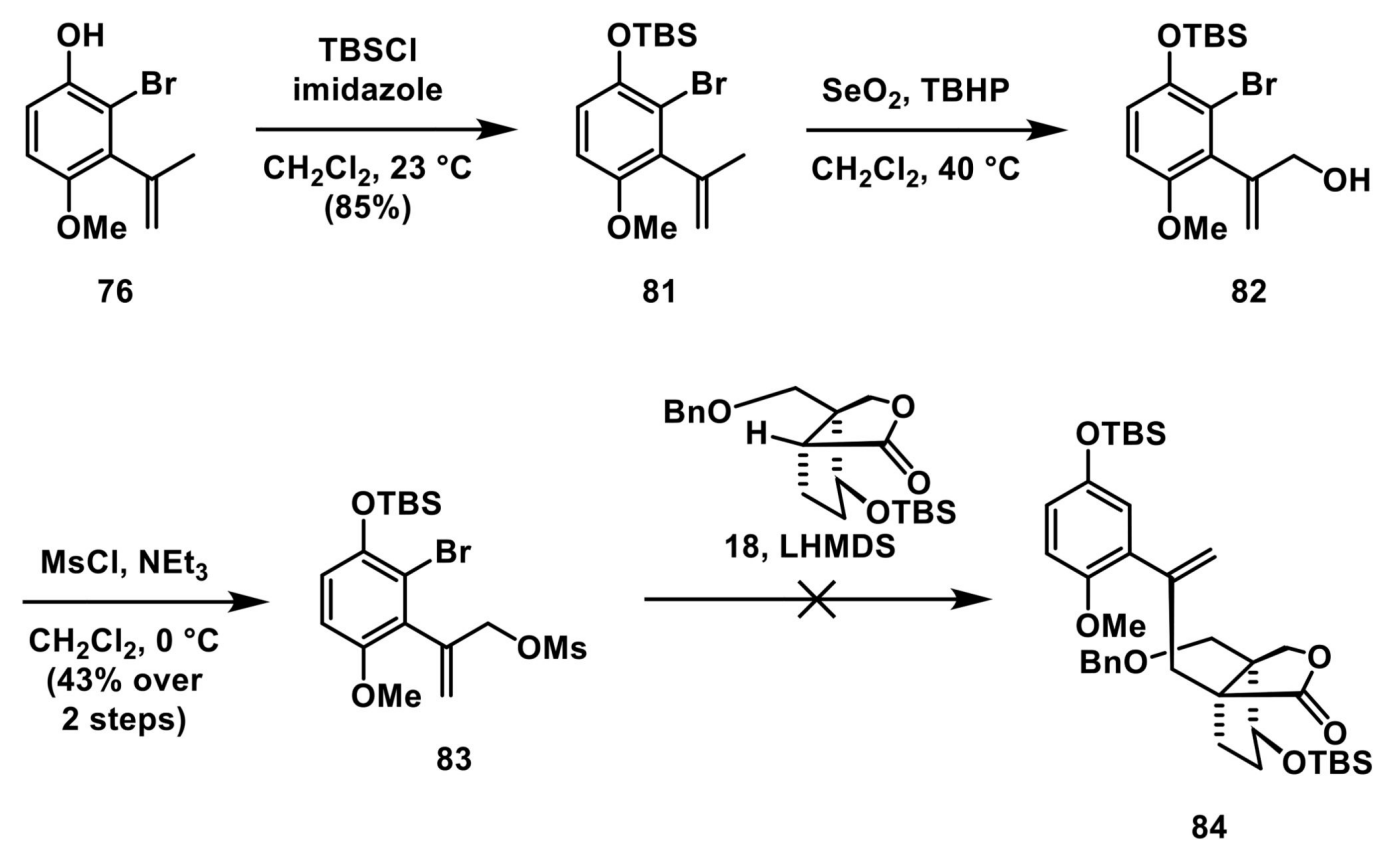
Synthesis of mesylate **83** and attempted alkylation of **18**.

Since the preparation of **80** proved more challenging than anticipated, we decided to explore the feasibility of the alkylation strategy using a less functionalized aromatic electrophile (Scheme 9). We went back to phenol **76**, protected the phenolic hydroxyl group as a TBS ether, and oxidized the allylic position using modified Riley conditions (SeO_2_, *tert*-butyl hydroperoxide (TBHP), 40 °C)^[52]^ to obtain alcohol **82**. After successful mesylation, we proceeded with the planned alkylation of lactone **18**. To this end, we generated the enolate from **18** upon treatment with LHMDS followed by addition of **83**. Unfortunately, the resulting enolate (not shown) did not react with mesylate **83** over a range of different temperatures.

#### 2.2.4 Late-Stage Biaryl Formation

**Scheme 10:**
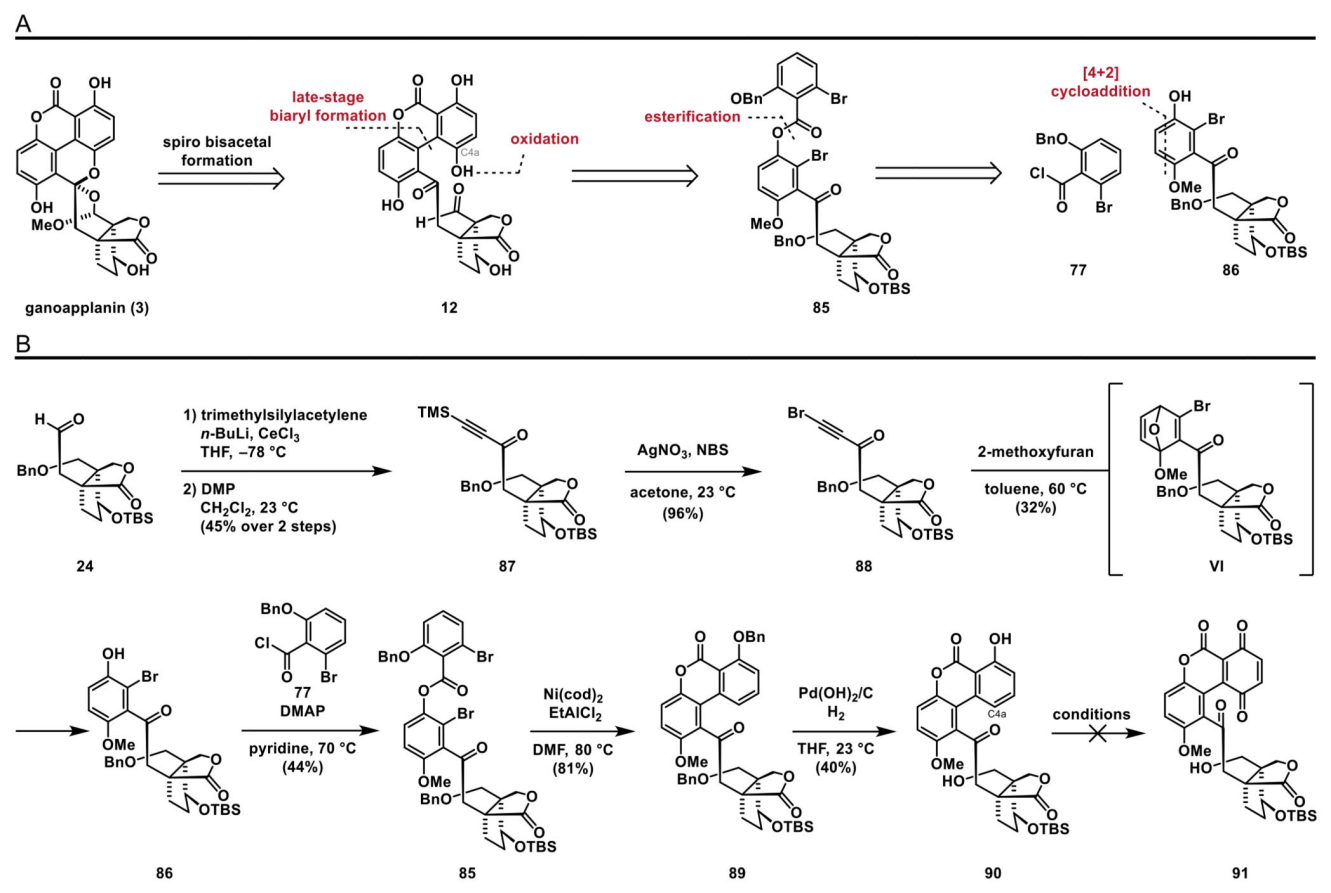
Retrosynthetic analysis and synthesis of biaryl **90**: A) retrosynthetic analysis for strategy **D** and B) synthesis of biaryl **90**.

Despite the various difficulties in merging the aromatic and terpenoid fragments, we achieved considerable success in synthesizing highly functionalized biaryl compounds using the Stille–Kelly cross-coupling described in the previous chapters. Building on this, we planned to implement this strategy as the key transformation in our next approach. Retrosynthetically, we envisioned biaryl **12** arising from an intramolecular cross-coupling reaction of bis-halogenated ester **85** (Scheme 10A). To access **85**, we planned to perform an esterification of acyl chloride **77** with phenol **86**. Based on our earlier approach, we decided to construct brominated phenol **86** through a Diels–Alder reaction between 2-methoxyfuran and a suitable brominated alkyne.

To install the alkyne moiety, we carried out a 1,2-addition of trimethylsilylacetylene, yielding a diastereomeric mixture of the corresponding secondary alcohol (not shown). Oxidation with Dess–Martin periodinane (DMP) converted this mixture to ketone **87** in 45% yield over two steps. The alkyne was brominated with *N*-bromosuccinimide (NBS), yielding an almost quantitative amount of bromo alkyne **88**. With bromo alkyne **88** in hand, we investigated the [4 + 2]-cycloaddition. To our satisfaction, heating **88** with 2-methoxyfuran at 60 °C gave phenol **86** in acceptable yields, probably via intermediate **VI**, which aromatizes spontaneously. The observed regioselectivity of the cycloaddition is in accordance with reported findings from Sonoda and is attributed to the electronic effects of the bromo substituent.^[40]^ Esterification was subsequently achieved by treating **86** with acyl chloride **77**, DMAP, and pyridine at elevated temperatures, yielding bis-halogenated ester **85**. With synthetic access to ester **85**, we were eager to attempt the envisioned Stille–Kelly cross-coupling. To our surprise, the previously used conditions (Pd(PPh_3_)_4_, Sn_2_Bu_6_, 140 °C) resulted only in a complex mixture, while conditions reported by Cramer (NiCl_2_(PPh_3_)_2_, Zn, 100 °C)^[54]^ failed to consume ester **85**. Fortunately, using Hosokawa’s conditions (Ni(cod)_2_, EtAlCl_2_, 80 °C)^[55]^ successfully provided biaryl **89** in high yield (81%).

Having constructed the crucial aryl–aryl bond, we next set out to investigate oxidation at the C4a position to form the hydroquinone motif. For this, we performed a global debenzylation (Pearlman’s catalyst Pd(OH)_2_/C, H_2_, 1 atm), which afforded phenol **90** (Scheme 10B). To install the missing C4a oxidation, we planned to oxidize the phenol moiety to its corresponding quinone **91**, followed by reduction to the corresponding hydroquinone.

To achieve the oxidation of compound **90** to the quinone, we screened several commonly used oxidants, such as PIDA, CAN, salcomine/O_2_, Fremy’s salt (potassium nitrosodisulfonate)^[56]^ and manganese(III) acetate.^[57]^ Unfortunately, those attempts resulted in no reaction (see the Supporting Information for details).

When **90** was subjected to PCC, the phenol moiety remained unaffected, while the primary alcohol was oxidized to the corresponding aldehyde (not shown). We hypothesized that the electron-withdrawing lactone group decreases the reactivity of the phenol moiety toward oxidation, thus impeding the desired transformation. Literature reports for phenol oxidation with an *ortho*-substituted carbonyl group are scarce, indicating that our target reaction may indeed be challenging to accomplish. To address this issue, we revisited earlier steps in our synthetic approach and decided to modify the intramolecular coupling precursor **85**.

**Scheme 11:**
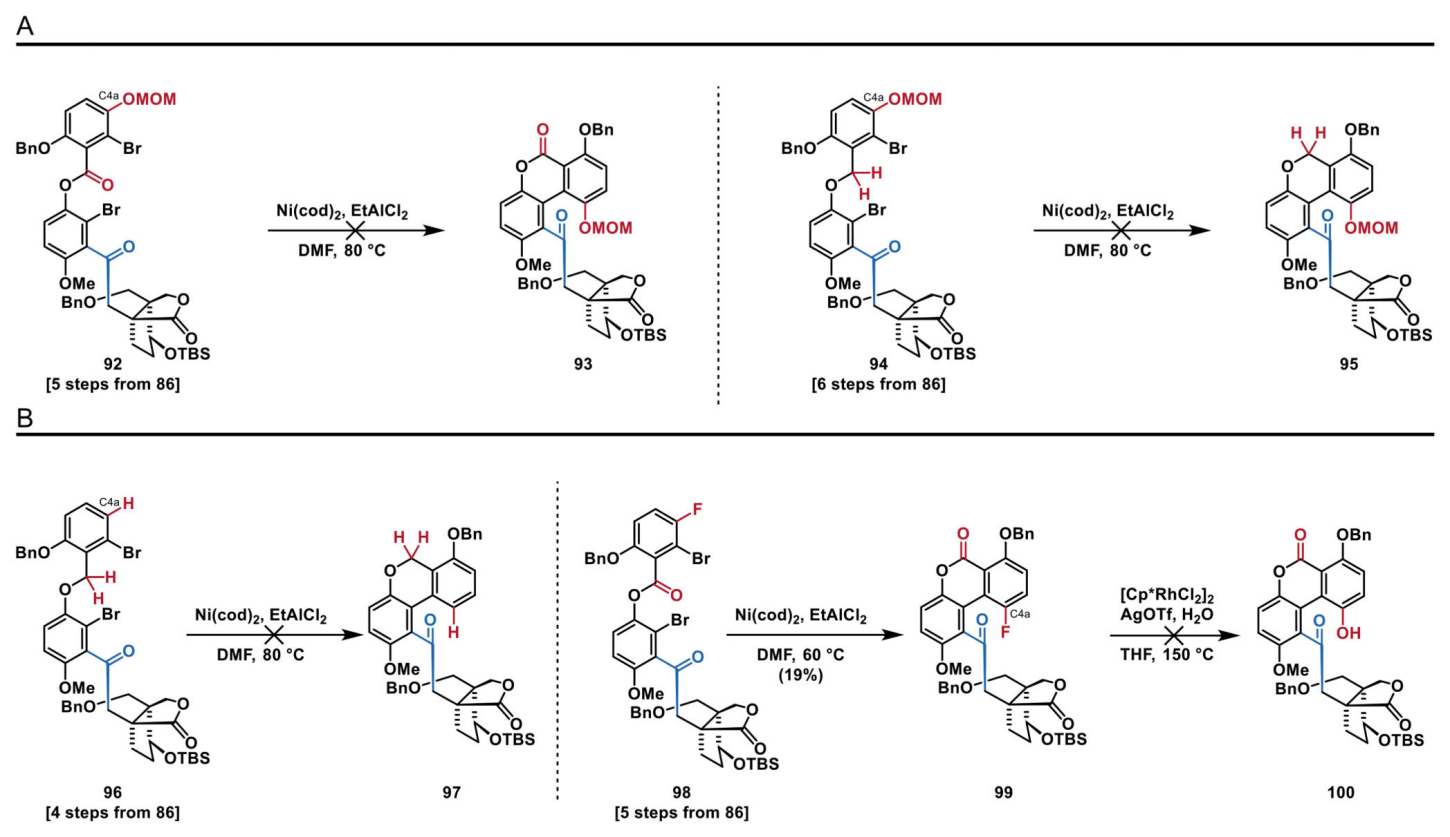
Intramolecular coupling attempts: A) attempted syntheses of **93** and **95** via intramolecular couplings and B) attempted intermolecular coupling of ether **96** and synthesis of fluorinated biaryl **99**.

Therefore, we synthesized ester **92** and ether **94** (see the Supporting Information for details), which already include the additional phenol group at C4a. Unfortunately, when treating either **92** or **94** with Ni(cod)_2_ and EtAlCl_2_ at 80 °C, we only observed decomposition (Scheme 11A). We attributed this outcome to increased steric hindrance from the additional substituent at C4a, which created a system with four *ortho* substituents around the reactive sites, making cross-coupling reactions particularly challenging. Similarly, subjecting ether **96** (see the Supporting Information for details) to the same conditions (Scheme 11B) led to formation of a complex mixture.

Additionally, we also envisioned the synthesis of fluorinated biaryl **99**, which could be a suitable substrate for an S_N_Ar reaction to introduce the hydroxy group at C4a. The intramolecular coupling reaction of **98** (see the Supporting Information for details) furnished the desired aryl fluoride **99** in low yields. For this specific reaction it was also important to lower the temperature from 80 to 60 °C as we observed decomposition otherwise. With this substrate in hand, we then set out to attempt the S_N_Ar reaction. Given that these types of reactions typically require harsh reaction conditions^[58]^ or are limited to electron-deficient arenes, we opted for a rhodium catalyzed S_N_Ar hydroxylation. Unfortunately, conditions reported by Shi,^[59]^ which proved to be applicable for the synthesis of electron-rich phenols, were found to be incompatible with our substrate, as we observed the formation of a complex mixture when aryl fluoride **99** was treated with [Cp*RhCl_2_]_2_ and water at 150 °C.

#### 2.2.5 Nucleophilic Addition

**Scheme 12:**
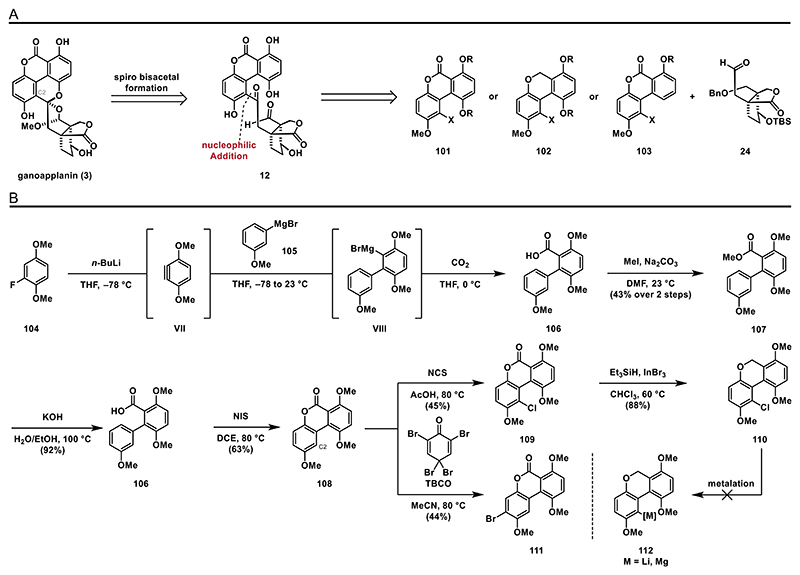
Retrosynthetic analysis and attempted preparation of organometallic compound **112**: A) retrosynthetic analysis for strategy E and B) synthesis of halogenated biaryls **109** and **111**.

Due to the difficulties encountered in realizing the oxidation of phenol **90** or forming the biaryl bond in compounds **93, 95**, or **97**, we decided to explore an alternative approach. Since we successfully achieved a 1,2-addition with metalated trimethylsilylacetylene to the terpenoid fragment, we based our next key transformation on this reactivity. Specifically, we aimed to prepare aryl halides **101, 102**, or **103**, which would undergo halogen/metal exchange followed by a nucleophilic 1,2-addition to aldehyde **24** to give access to **12** (Scheme 12A). Since **101** and **102** feature tetra-*ortho*-substituted biaryl bonds, typically challenging to form through transition metal-catalyzed cross-coupling reactions, we sought an alternative strategy. Inspired by the work of Barrett^[60]^ and Buchwald,^[61]^ we envisioned a three-component reaction to form these biaryls, utilizing aryne intermediate **VII**. Treatment of 2-fluoro-1,4-dimethoxybenzene (**104**) with *n*-BuLi followed by addition of the Grignard reagent **105** (prepared from commercially available 3-bromoanisole, not shown) led to formation of **VIII**, which was subjected to carbon dioxide to yield benzoic acid **106**. To simplify the purification by column chromatography on silica gel, we converted the acid **106** to methyl ester **107** (43% over 2 steps). Subsequent saponification followed by oxidative lactonization, under conditions reported by Wei (*N*-iodosuccinimide (NIS), 75 °C),^[62]^ afforded the desired biaryl lactone **108** in 63% yield. Alternative conditions such as PIDA/Pd(OAc)_2_^[63]^ or K_2_S_2_O_8_/AgNO_3_^[64]^ only led to decomposition. Photochemical (CeCl_3_, 419 nm)^[65]^ and electrochemical conditions (Pt electrodes, 23 mA, 5 V)^[66]^ also failed to form the desired product **108**.

With a synthetic route to compound **108** established, we then focused on halogenating the C2 position. Attempted bromination using NBS failed to react at 23 °C and produced complex mixtures at elevated temperatures. Using 2,4,4,6-tetrabromo-2,5-cyclohexadienone (TBCO) as the bromine source yielded the undesired regioisomer **111** in 44% yield. Chlorination of the C2 position, however, proved more successful: treatment with *N*-chlorosuccinimide (NCS) in acetic acid at 80 °C gave aryl chloride **109** as the only isolable regioisomer in 45% yield. We hypothesize that the C2 position is indeed the most reactive site for electrophilic halogenation but may be sterically too hindered for bromination (Scheme 12B).

To fuse aryl chloride **109** and aldehyde **24** we planned a metalation, followed by nucleophilic 1,2-addition.

Unfortunately, attempts to form the corresponding organometallic intermediate by treatment of **109** with magnesium, magnesium and lithium chloride,^[67]^ zinc and lithium chloride, lithium or 4,4’-di-*tert*-butyldiphenylide (LiDBB)^[68,69]^ failed and either decomposition or no reaction was observed (see the Supporting Information for details). Attempts of a halogen/metal exchange using either *n*-BuLi or *t*-BuLi resulted in complex mixtures. Additionally, the lactone moiety of **109** may be incompatible with nucleophilic organolithium reagents like *n*-BuLi or *t*-BuLi. To address this, we chose to reduce the lactone moiety in **109** by treating it with triethylsilane and catalytic indium(III) bromide,^[70]^ yielding the cyclic ether **110** in 88% yield. With this substrate in hand, we set out to investigate the metalation as well; however, in this case neither treatment with magnesium, LiDBB, *n*-BuLi or *t*-BuLi led to formation of **112**, and only unreacted starting material was recovered.

**Scheme 13:**
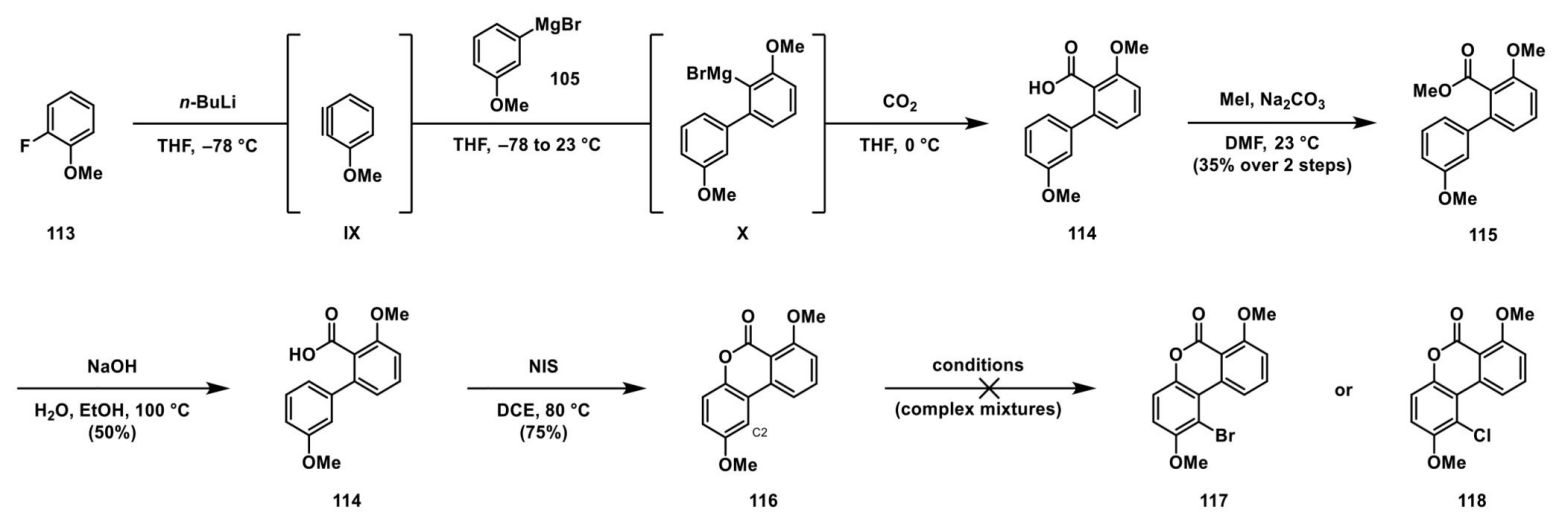
Synthesis of biaryl **116** and halogenation attempts.

To reduce steric hindrance, we synthesized a less encumbered biaryl compound **116** by slightly adapting our established synthetic route. Starting from commercially available 1-fluoro-3-methoxybenzene (**113**), we generated the corresponding benzyne intermediate **IX**, which was subsequently treated in situ with Grignard reagent **105** and carbon dioxide. This yielded crude benzoic acid **114**, which was transformed into methyl ester **115** with an overall yield of 35% over two steps to simplify purification by column chromatography. Saponification afforded pure benzoic acid **114**, which was then treated with NIS to induce oxidative lactonization, to lactone **116**. With this substrate in hand, we screened various conditions for halogenation at the C2 position. However, under all investigated conditions, we observed exclusively the formation of undesired regioisomers (Scheme 13).

### 2.2.6 Cationic Cyclization/Fries Rearrangement

**Scheme 14:**
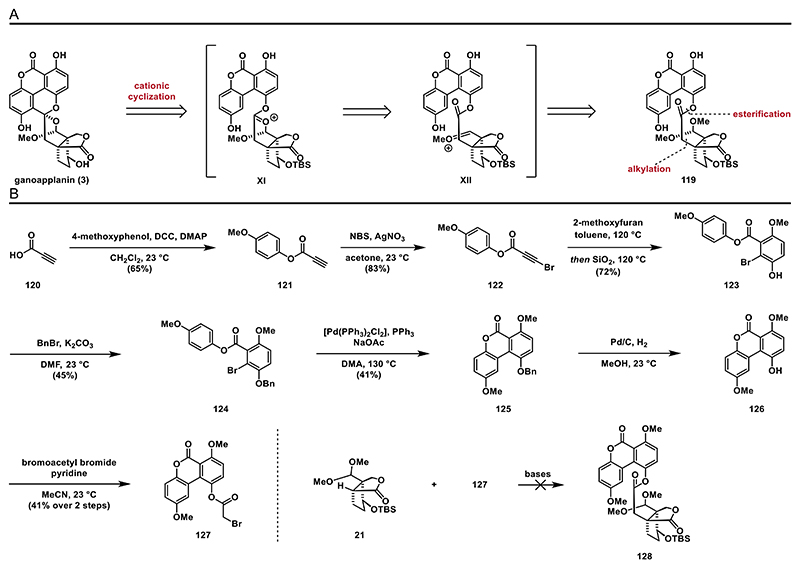
Synthesis of bromoester **127** and attempted alkylation of lactone **21**: A) retrosynthetic analysis for strategy F and B) synthesis of phenol **126**.

After reaching a dead end, we decided to reconsider our strategy and design a new strategy involving a cationic cyclization. We envisioned that exposure of ester **119** to a Lewis or Brønsted acid would activate the acetal group and generate the oxocarbenium ion **XI** and **XII**. This would then trigger a nucleophilic attack of the benzene ring, leading to the formation of the spiro bisacetal structure (Scheme 14A).

To investigate this key step, we initially set out to synthesize α-bromoester **127** and then attach it to lactone **21** via deprotonation followed by alkylation. We commenced our synthetic endeavor with an initial esterification step (*N*,*N*′-dicyclohexylcarbodiimide (DCC), DMAP) between propiolic acid (**120**) and 4-methoxyphenol to yield ester **121**. The terminal alkyne was brominated employing silver nitrate and NBS to afford bromoalkyne **122** (Scheme 14B). When alkyne **122** was treated with 2-methoxyfuran, a cycloaddition adduct was formed and subsequently transformed into bromobenzoate **123** upon exposure to silica gel.^[40]^ The hydroxyl group was then protected as a benzyl ether to yield **124**. To form the tricyclic structure, various C–H activation conditions were screened: We found that exposure of **124** to Pd(PPh_3_)_2_Cl_2_, triphenylphosphine, and sodium acetate in dimethyl acetamide (DMA) at 130 °C furnished desired product **125** in 41% yield.^[71]^ Hydrogenolysis of the benzyl group, followed by esterification of phenol **126** with bromoacetyl bromide, yielded bromoester **127**. Unfortunately, an attempt to alkylate lactone **21** with α-bromoester **127** was unsuccessful. While the lactone **21** remained intact under the reaction conditions, the α-bromoester **127** decomposed. It is likely that **127** is more acidic than **21**, resulting in its deprotonation by the enolate generated from lactone **21**. The resulting anion may undergo decomposition pathways.

To overcome the seemingly challenging alkylation step, we aimed for a synthesis of phenol **133** and carbonylimidazole **135** and a subsequent esterification to access **136**.

**Scheme 15:**
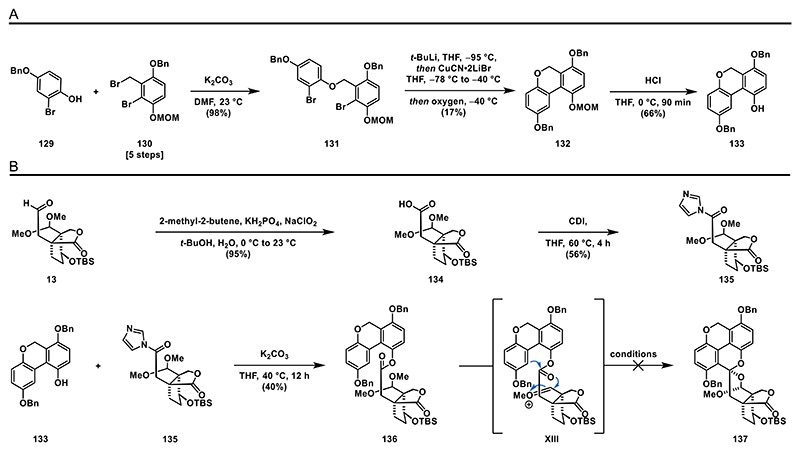
Attempted cationic cyclization: A) synthesis of phenol **133** and B) synthesis of amide **135** and ester **136**.

We commenced our synthetic approach toward phenol **133** with the formation of bis-brominated ether **131** by treatment of a mixture of benzyl bromide **130** (see the Supporting Information for details) and phenol **129** with potassium carbonate. After screening various conditions for the crucial aryl–aryl bond formation, we found that Lipshutz’s conditions afforded the desired biaryl **132** in acceptable yields.^[72,73]^ The MOM-protected phenol was then deprotected with aqueous hydrochloric acid, yielding phenol **133** (Scheme 15A).

With the synthesis of **133** completed, we turned our attention to the formation of ester **136**. Aldehyde **13** was oxidized to the corresponding carboxylic acid **134** using the Pinnick–Lindgren–Kraus oxidation (Scheme 15B).^[74]^ To form **136**, we activated carboxylic acid **134** using Staab’s reagent (CDI) under basic conditions to obtain carbonylimidazole **135**^[75]^ and treated it with phenol **133** to give ester **136** in a 40% yield.

With the cyclization precursor **136** prepared, we tested several Lewis and Brønsted acids to initiate the cationic cyclization. However, we only observed deprotection of the acetal and desilylation upon exposure to various acids. Notably, this result was unaffected by the substituents on the hydroxyl groups, as a debenzylated intermediate also failed to form the desired product by treatment with Lewis and Brønsted acids (see the Supporting Information for details). As a result of the failure of this approach, we decided to slightly modify our synthetic strategy.

**Scheme 16:**
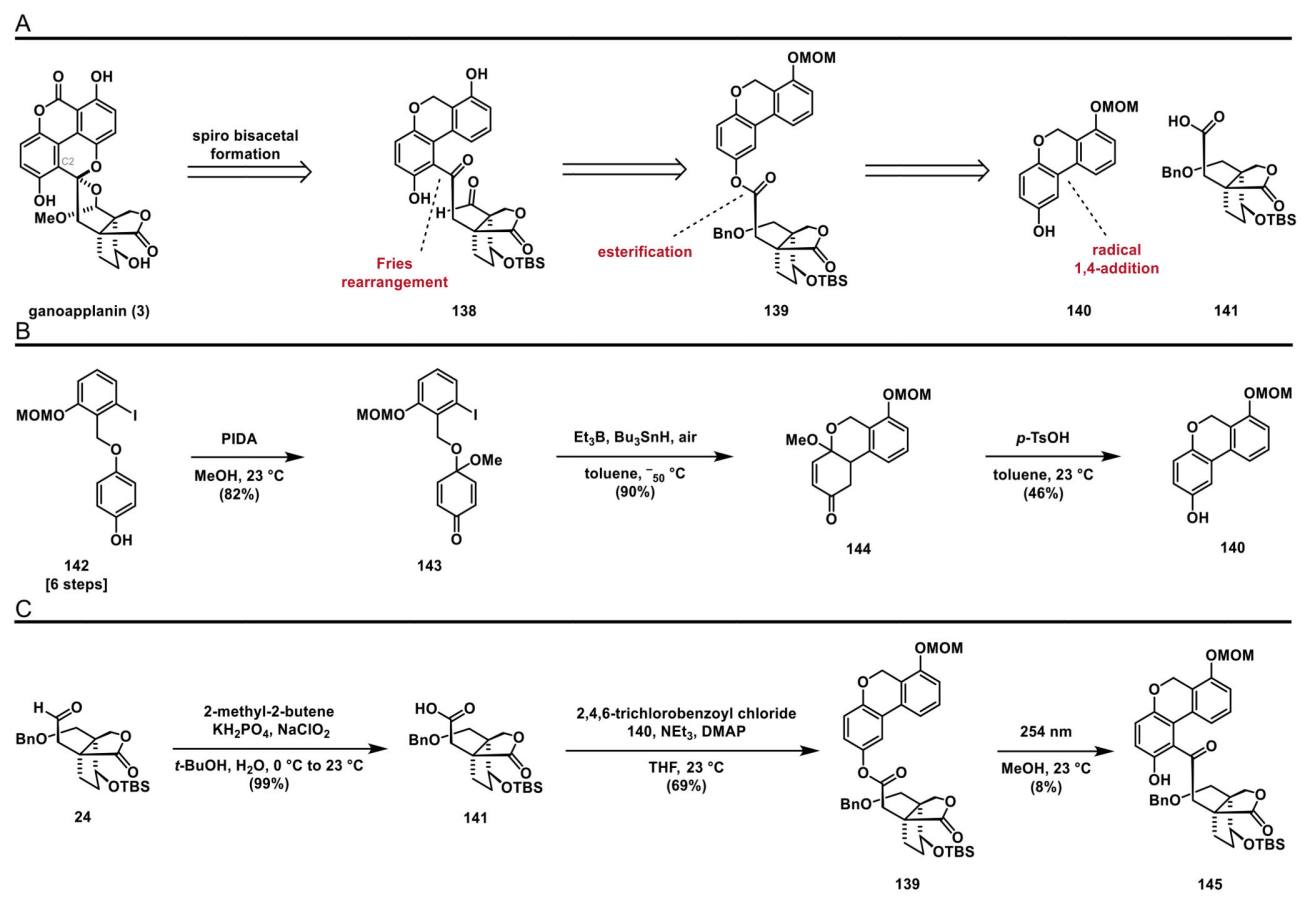
Fries rearrangement approach: A) retrosynthetic analysis for strategy **F**; B) synthesis of phenol **140**; and C) Fries rearrangement.

Given our recent success in synthesizing related GMs through a Fries rearrangement as the pivotal step, we chose to apply this method to ganoapplanin (**3**) as well.^[19]^ This approach led us to trace key intermediate **138** back to ester **139** (Scheme 16A), which should be attainable via the esterification of phenol **140** with carboxylic acid **141**.

To access phenol **140** we employed an oxidative dearomatization of phenol **142** (see the Supporting Information for details) using (diacetoxyiodo)benzene (PIDA), affording quinone monacetal **143** in 82% yield^[76]^ (Scheme 16B). To construct the aryl–aryl bond, we then explored various conditions to facilitate an intramolecular 1,4-addition of aryl iodide **143** to its enone system (**Table 1**). Treatment of **143** with *t*-BuLi at -78 °C resulted in a complex mixture (entry 1). Adding hexamethylphosphoramide (HMPA) or *N*,*N*′-Dimethylpropyleneurea (DMPU) did not change the reaction outcome (entries 2 and 3). Attempts to generate an organomagnesium intermediate for 1,4-addition, using Turbo–Grignard (*i-*PrMgCl·LiCl) at 0 °C also resulted in the decomposition of **143** (entry 4).

Shifting away from anionic conditions, we then explored radical conditions and found that treating **143** with AIBN and tributyltin hydride at elevated temperatures yielded tricycle **144** in high yield (entry 5).^[77]^ Further optimization by using triethylborane (BEt_3_) and tributyltin hydride ((*n-*Bu_3_)SnH) improved the reaction, providing the desired product **144** as a single diastereomer in 90% yield (entry 6).^[78]^ Enone **144** was then aromatized by treatment with *p*-TsOH at 23 °C affording phenol **140**.^[79]^

With the successful synthesis of phenol **140**, we shifted our focus to the preparation of ester **139** (Scheme 16C). This was achieved by converting aldehyde **24** into carboxylic acid **141** under Pinnick–Lindgren–Kraus conditions, followed by treatment with Yamaguchi’s reagent,^[80]^ **140**, and NEt_3_, which afforded ester **139** in 69% yield.

With ester **139** in hand, we proceeded to screen conditions for the key Fries rearrangement to afford **145** (**Table 2**).^[81]^ Treatment with Lewis acids such as (Sc(OTf)_3_, AlCl_3_, BF_3_.OEt_2_; entries 1–3) resulted in no reactions at lower temperatures (<100 °C) and decomposition at 150 °C. We reasoned that conditions employing Lewis acids might be too harsh for our substrate. Building on our prior work using photochemical conditions for Fries rearrangements,^[19]^ we irradiated **139** at 254 nm.^[82]^

In acetonitrile or cyclohexane, only complex mixtures were formed (entries 4 and 5), while irradiating **139** in methanol gave the desired product **145** in ≈8% yield (entry 6).

Unfortunately, we were unable to establish a protocol to obtain ketone **145** in synthetically useful yields, leading us to abandon this approach. However, the remarkable efficiency of the triethylborane-mediated radical 1,4-addition of aryl iodide **143** to form the biaryl motif captured our interest. Thus, we decided to further explore the potential of incorporating this step into the total synthesis of ganoapplanin (**3**). The results of those investigations are presented in the next section.

#### 2.2.7 Radical 1,4-Addition/Aldol Sequence

**Scheme 17:**
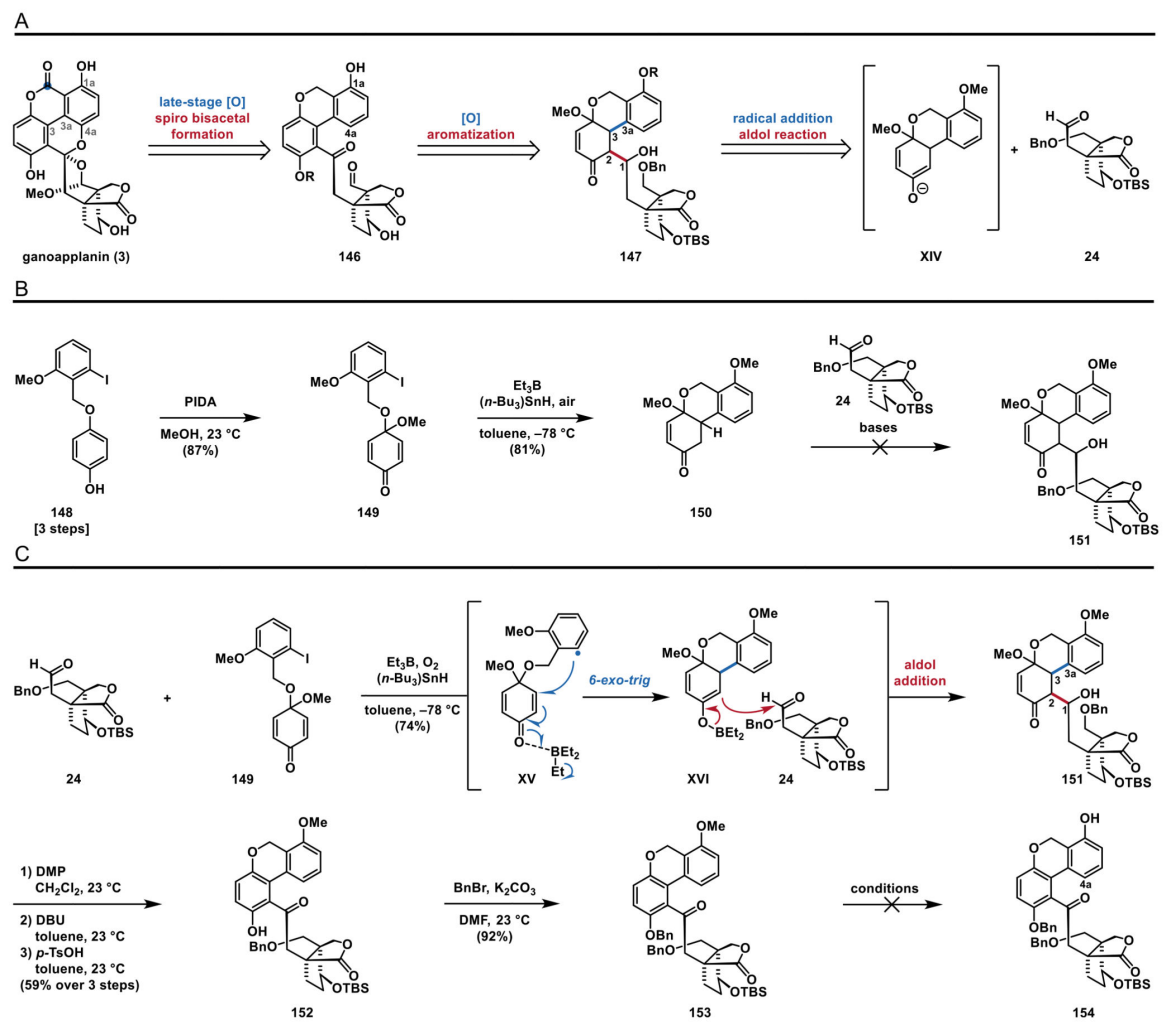
Retrosynthetic analysis and fusion of the components: A) retrosynthetic analysis for strategy G; B) attempted aldol addition; and C) radical 1,4-addition/aldol cascade.

Building on the findings described in the previous section, we devised a new strategy to access ganoapplanin (**3**). For this purpose, we traced **3** back to phenol **146** through late-stage oxidation and spiro bisacetalization. **146** could arise from ß-hydroxyketone **147** via aromatization. To construct **147**, we envisioned an intermolecular aldol addition of enolate **XIV** and aldehyde **24** (Scheme 17A). To investigate this strategy, we set out to synthesize phenol **148** (see the Supporting Information for details), which was subsequently oxidatively dearomatized to form quinone monoacetal **149**. Radical initiation with triethylborane, tributyltin hydride, and air generated the corresponding aryl radical, which underwent an intramolecular 1,4-addition to form enone **150** (Scheme 17B).

With enone **150** in hand, we proceeded to screen conditions for the aldol addition to aldehyde **24** to form hydroxyketone **151**. Surprisingly, treatment with bases like LDA, KHMDS, or LHMDS failed to consume the starting materials at both low temperatures (−78 °C) and 23 °C. Attempts to generate boron enolates^[83]^ using either *n*-Bu_2_BOTf or Cy_2_BOTf also did not yield hydroxyketone **151** (see the Supporting Information for details). As our envisioned two step sequence including an intramolecular radical 1,4-addition followed by an intermolecular aldol addition failed, we took a closer look into literature. Reports by Utimoto^[84]^ and Inoue^[85]^ describe the triethylborane-mediated radical 1,4-additions of alkyl radicals into enones, followed by in situ boron enolate formation and aldol additions to aldehydes. Inspired by these intriguing results, we decided to apply these conditions to our substrates **24** and **149**.

We ultimately found that radical initiation using triethylborane and oxygen in the presence of tributyltin hydride successfully induced both the intramolecular radical 1,4-addition of **149** and the intermolecular aldol reaction with aldehyde **24**, yielding **151** as a mixture of diastereomers in 74% yield. Triethylborane serves a dual function in this process: (1) initiating the radical reaction to enable the 6-*exo*-trig cyclization of aryl radical **XV**,^[86]^ and (2) forming boron enolate **XVI**, which facilitates the aldol addition with **24**.^[84,85]^ This sequence efficiently joined both fragments in high yield, establishing the key C3−C3a and C1−C2 bonds in a single step. Notably, the presence of both triethylborane and tributyltin hydride was essential, as omitting either reagent resulted in no desired reactivity (Scheme 17C). To construct the biaryl motif, we proceeded with the aromatization of enone **151**. This sequence started by oxidizing the secondary alcohol to the ketone using DMP, followed by treatment with *p*-toluenesulfonic acid (*p*-TsOH) to obtain **152** in 80% yield. However, this method was inconsistent and difficult to reproduce. While optimizing this process, we discovered that exposing the formed ketone to 1,8-diazabicyclo[5.4.0]undec-7-ene (DBU) generated a mixture of diastereomers, which then underwent smooth aromatization when treated with *p*-TsOH. We then continued our synthetic route with a benzyl protection of phenol **152** yielding benzyl ether **153** almost quantitatively (92%).

To achieve the C4a oxidation and form the hydroquinone motif, we envisioned an oxidation of phenol **154** to its quinone. Therefore, we aimed for a selective deprotection of the methoxyether, which, unfortunately, turned out to be very challenging. Initial screening of conditions, including BiCl_3_ at −78 °C and Ph_2_PLi^[87]^ at 70 °C, led to the liberation of the benzyl protected phenol through debenzylation. Treatment with either BBr_3_ or AlCl_3_ and *t*-BuSH, however, resulted in decomposition. When **153** was subjected to LiCl^[88]^ or Na_2_S^[89]^ at elevated temperatures, no reaction was observed (see the Supporting Information for details).

**Scheme 18:**
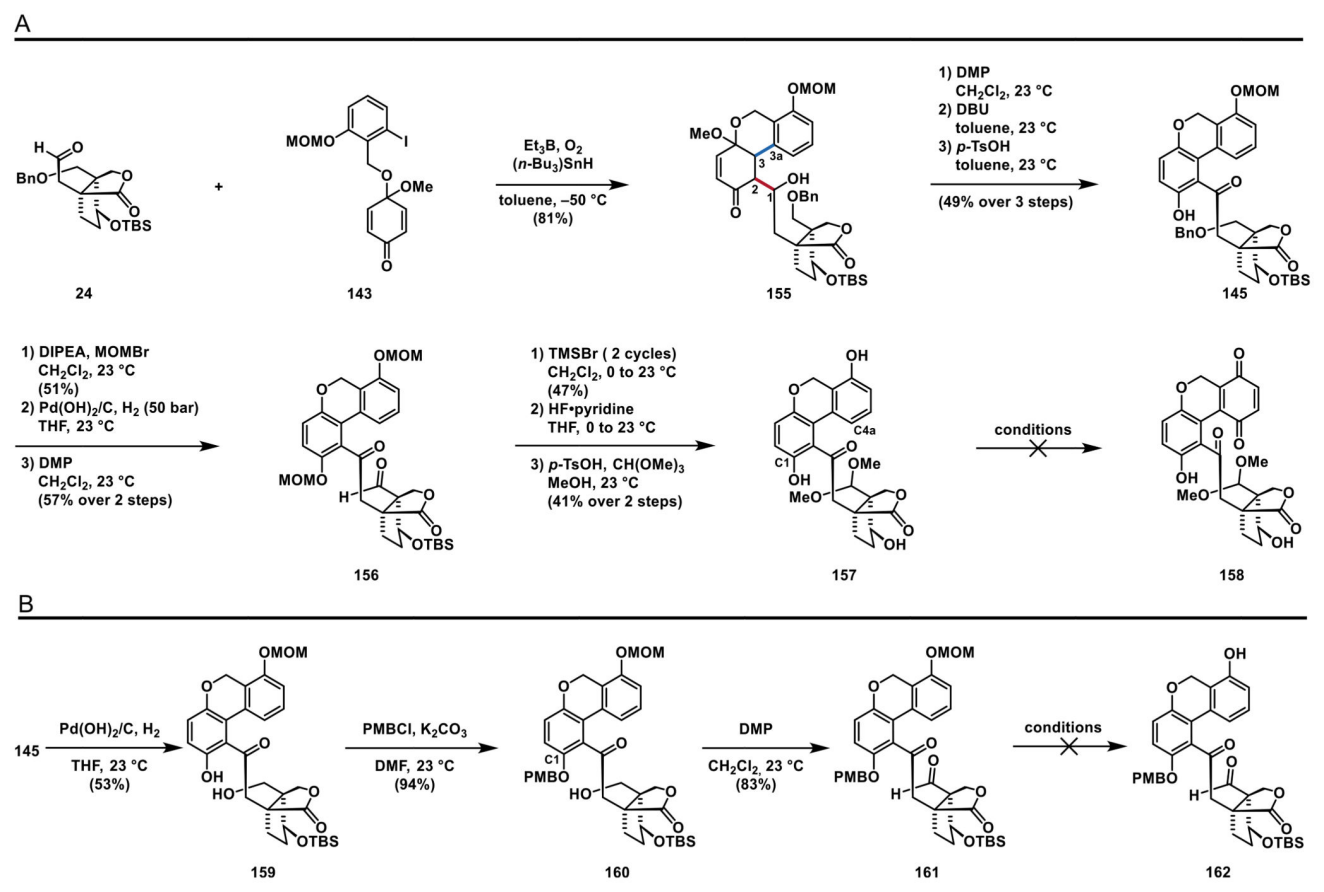
Sequence leading to bisphenol **157** and synthesis of aldehyde **161**: A) synthesis of phenol **157** and B) synthesis of aldehyde **161**.

Since we were unable to remove the protecting group from **153**, we slightly modified our key radical addition/aldol reaction cascade, opting to use quinone monoacetal **143** with a MOM protected phenol that could be removed in later stages (Scheme 18A). To our satisfaction, the key cascade reaction with aldehyde **24** and quinone monoacetal **143** proceeded smoothly, yielding hydroxyketone **155** in 81%. Next, we oxidized the secondary alcohol to the corresponding ketone (not shown) and carried out the aromatization of the enone moiety under previously used conditions (DMP, DBU, and *p*-TsOH) to obtain biaryl **145** in 49% yield. This yield was somewhat lower than that of biaryl **152** (80% over three steps), likely due to the acid sensitivity of the MOM protecting group.

To continue, the unprotected C1 phenol group was masked as a MOM ether, the benzyl ether was cleaved from the primary and subsequently oxidized to aldehyde **156** using DMP. The following cleavage of the MOM ethers was realized by treatment with an excess of TMSBr and was followed by desilylation using HF·pyridine and dimethylacetalization of the aldehyde moiety using *p*-TsOH, CH(OMe)_3_, and methanol.

With bis-phenol **157** synthesized, we proceeded to attempt the C4a oxidation. Using hypervalent iodine reagents, such as (bis(trifluoroacetoxy)iodo)benzene (PIFA) and PIDA, only led to complex mixtures. Other commonly used quinone-forming oxidants, including Fremy’s salt, CAN, salcomine/O_2_, K_2_CO_3_/O_2_, and CrO_3_, showed no reactivity, as **157** was not consumed in any of these attempts (see the Supporting Information for details).

From these C4a oxidation attempts, we concluded that a protecting group on the C1 phenol is necessary to prevent undesired side reactions. We opted for a PMB protecting group and treated phenol **159** with PMBCl and K_2_CO_3_ leading to ether **160** in nearly quantitative yield. Oxidation to aldehyde **161** was achieved using DMP (83% yield). However, attempts to cleave the MOM ether proved challenging due to the greater lability of the PMB protecting group on the C1 phenol. Conditions involving either Lewis or Brønsted acids selectively removed the PMB group or simultaneously cleaved both the PMB and MOM protecting groups, ultimately yielding phenol **157** (Scheme 18B).

**Scheme 19:**
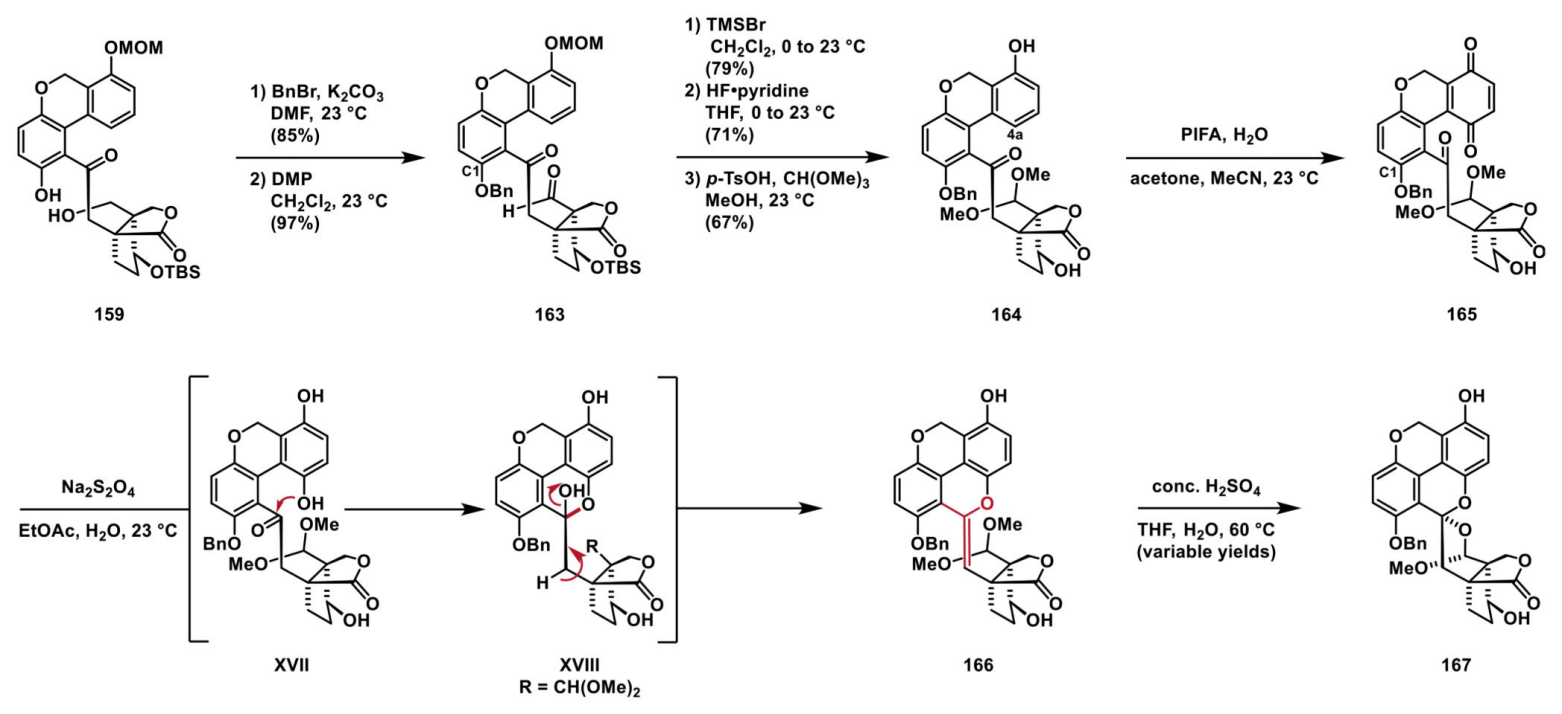
Synthesis of acetal **165**.

We were then keen to investigate the usability of a benzyl protecting group on the C1 phenol. For this purpose, we conducted a chemoselective benzylation of the phenol group of **159**, followed by the oxidation of the primary alcohol to corresponding aldehyde **163**. At this stage, we removed the MOM and TBS protecting groups through sequential treatment with TMSBr^[90]^ and HF·pyridine and the aldehyde group was then transformed into a dimethyl acetal under acidic conditions, providing **164** in 67% yield. For the critical oxidation of the C4a position, we converted the phenol to the corresponding quinone **165**, which was immediately reduced with sodium dithionite. Unexcitedly, the spiro bisacetal structure was not formed under these conditions. Instead, we identified enol ether **166** as the sole component in the crude ^1^H-NMR. Unfortunately, enol ether **166** was unstable on silica gel, making isolation challenging. We assume **166** forms through the elimination of the corresponding hemiacetal **XVIII**. Subjecting **166** to concentrated sulfuric acid at elevated temperatures (60 °C) produced trace amounts of spiro bisacetal **167** (Scheme 19). We were surprised to find that the alcohol group of the formed hemiacetal **XVIII** did not react with the dimethyl acetal to yield the desired spiro bisacetal structure. We hypothesize that this outcome may be due to steric hindrance from the benzyl protecting group on the C1 phenol. To test this hypothesis, we attempted the C4a oxidation using an acetyl protecting group at the C1 phenol.

**Scheme 20:**
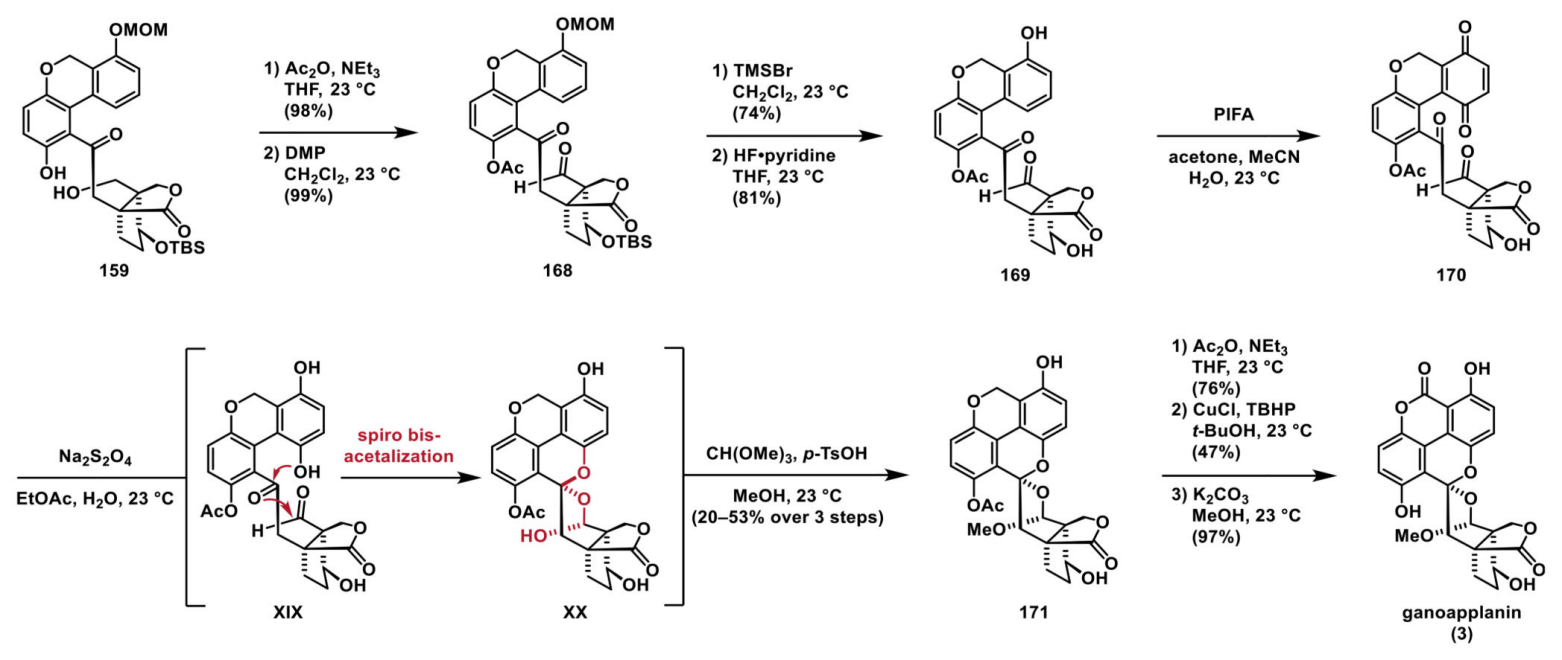
Synthesis of ganoapplanin (3).

Phenol **159** was treated with acetic anhydride and triethylamine to give the corresponding acetyl ester, which was then subjected to DMP, which smoothly oxidized the primary alcohol to aldehyde **168** in 99% yield.^[28]^ The MOM and TBS protecting groups were subsequently removed using TMSBr and HF·pyridine to give phenol **169**. We then turned our attention to the C4a oxidation followed by reductive spiro bisacetalization. Oxidation with PIFA generated quinone **170**, which proved to be unstable and was therefore used directly in the next step without purification. Treating a solution of crude quinone **170** in ethyl acetate with sodium dithionite resulted in a mixture of hydroquinone **XIX** and hemiacetal **XX**, which was then converted into spiro bisacetal **171** using trimethyl orthoformate and *p*-TsOH in methanol. Interestingly, in this case, we did not observe the elimination of water from the formed intermediate as we did not detect formation of the corresponding enol ether. To ensure efficient oxidation at the benzylic position, we protected the phenol moiety as its acetyl ester, followed by treatment with *tert*-butyl hydroperoxide and catalytic copper(I) chloride to yield the corresponding lactone.^[91]^ In the final step, the acetyl protecting groups were removed with potassium carbonate in methanol, completing the synthesis of ganoapplanin (**3**) (Scheme 20).

**Scheme 21:**
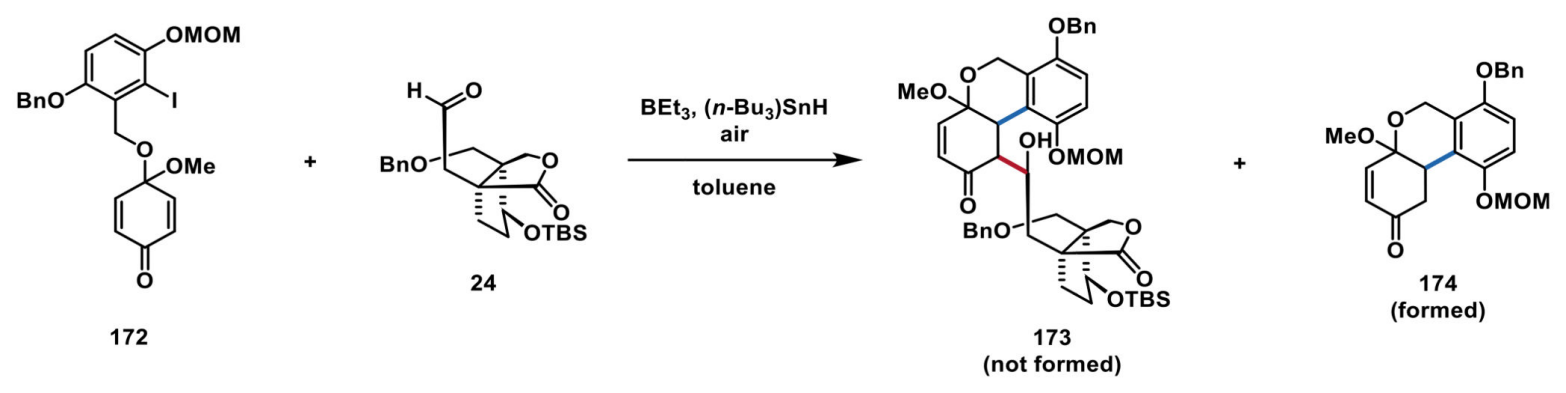
Attempted radical 1,4-addition/aldol sequence employing quinone monoacetal **172**.

This culminated in the first published total synthesis of the dimeric *Ganoderma* natural product, ganoapplanin (**3**), achieved in 25 steps (20 steps in the longest linear sequence).^[28]^ The spectroscopic data of synthetic ganoapplanin (**3**) were in full agreement with the literature values.^[4]^ To further enhance the efficiency of our synthetic sequence for ganoapplanin (**3**), we focused on improving the key radical 1,4-addition/aldol cascade. Using quinone monoacetal **143** in this cascade requires introducing the hydroquinone motif later in the synthesis via oxidation of phenol **169**. We wondered whether it would be possible to use a highly oxidized aromatic fragment, already containing the hydroquinone motif, for the cascade reaction, thereby eliminating the need for the C4a oxidation step. To explore this, we set out to synthesize quinone monoacetal **172** (see the Supporting Information for details). Unfortunately, when we attempted the radical 1,4-addition/aldol sequence with aldehyde **24** and quinone monoacetal **172** at −78 °C, the aldol product **173** did not form. However, we did observe the formation of tricycle **174**, indicating that the 1,4-addition step was successful, but the aldol addition failed. Increasing the reaction temperature to −50 or 0 °C did not change the outcome, while running the reaction at 23 °C led to decomposition of the starting materials (Scheme 21).^[92]^

## 3 Conclusion

**Scheme 22:**
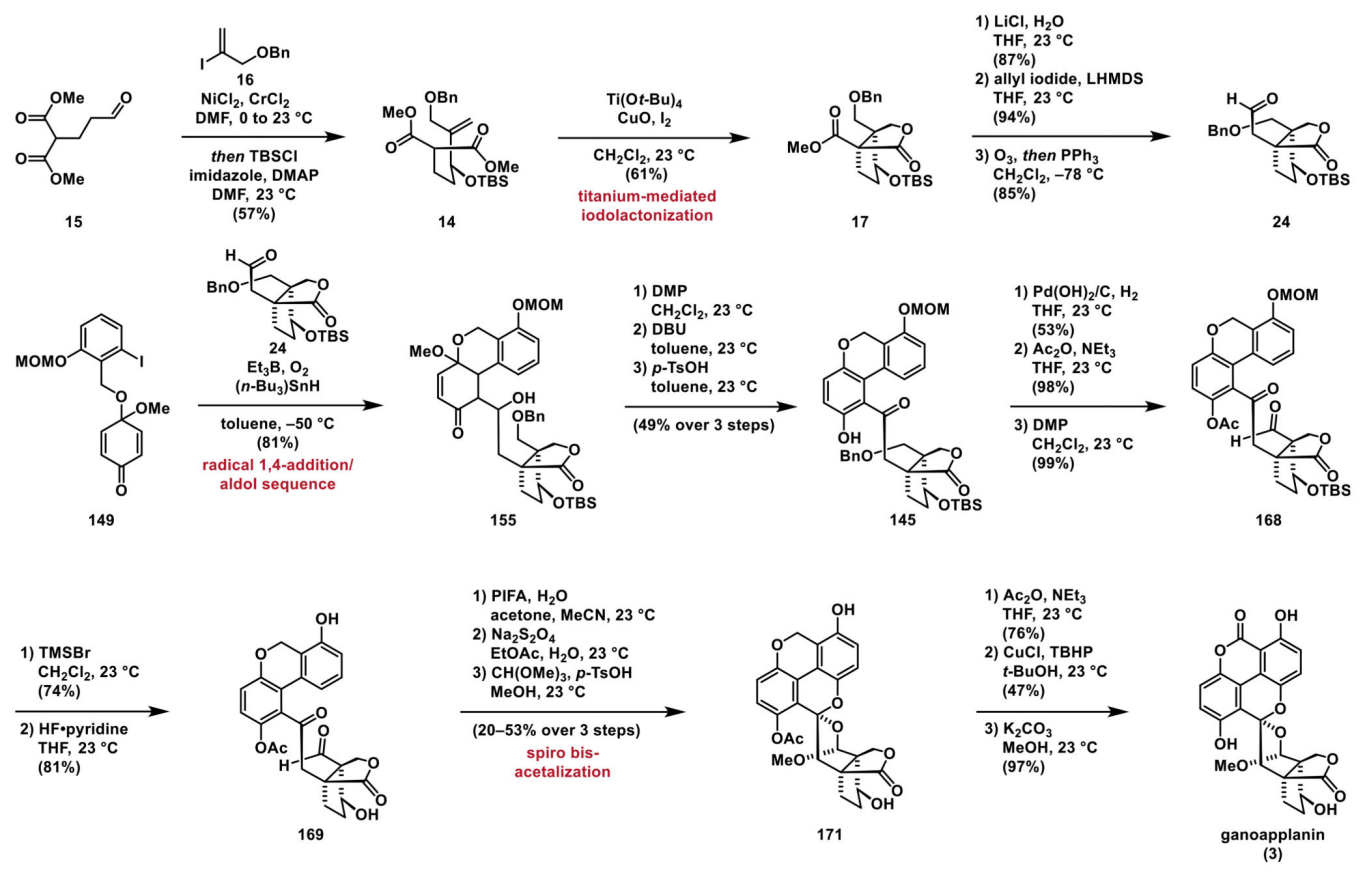
Synthetic route to ganoapplanin (3).

In conclusion, we have reported the development of the first synthetic access to the GM ganoapplanin (**3**). Our studies explored the convergent assembly of a range of aromatic fragments with the southern terpenoid component. A key step in constructing the latter was a highly efficient and diastereoselective titanium-mediated iodolactonization. Our initial strategy utilized two consecutive [4 + 2]-cycloadditions of a diyne motif with 2-methoxyfuran to generate the highly functionalized biaryl core. While the first cycloaddition was successful, attempts to achieve the second failed. We then pivoted to the synthesis of functionalized biaryl lactones, accessed through the Stille–Kelly reaction, and investigated several methods to link these to the southern terpenoid fragment. As these attempts were unsuccessful, we employed a nickel-mediated coupling to forge the aryl–aryl bond at a later stage, successfully synthesizing a biaryl lactone with the complete carbon skeleton of ganoapplanin (**3**). Unfortunately, attempts to install the missing phenol moiety at C4a via late-stage oxidations were unsuccessful. We next turned to halogenated biaryls, aiming to add them to the southern fragment via nucleophilic additions. This strategy involved the addition of an organomagnesium reagent to an aryne, followed by reaction with carbon dioxide to form a benzoic acid, which underwent oxidative lactonization. While selective chlorination of the biaryl lactone was achieved, subsequent metalation proved unfeasible. This led us to investigate new strategies based on Fries rearrangements and cationic cyclizations. Here, phenol-containing biaryls were esterified with the southern fragment, followed by a Lewis acid or light-mediated acyl migration. Unfortunately, these methods either resulted in decomposition or lacked efficiency (Scheme 16). A breakthrough was achieved with a two-component coupling strategy involving a 6-*exo*-trig radical addition of a quinone monoacetal, followed by an intermolecular aldol reaction cascade. This cascade simultaneously constructed the C3−C3a bond and fused the northern and southern fragments. Two late-stage oxidations then facilitated the critical C4a oxidation and the introduction of both the spiro bisacetal skeleton and the lactone moiety. This strategy culminated in the first total synthesis of ganoapplanin (**3**) (Scheme 22).

## Figures and Tables

**Table 1:** Screened reaction conditions for intramolecular 1,4-addition of **143**.

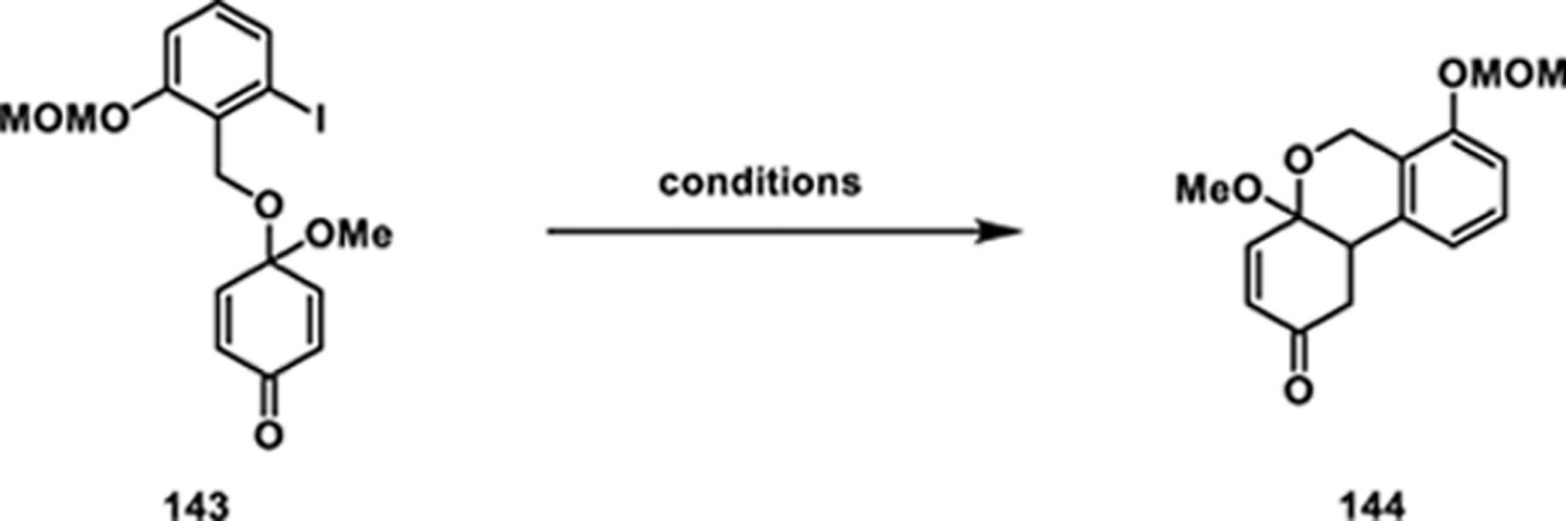				
Entry	Conditions	Solvent	*T* [°C]	Observation
1	*t*-BuLi	THF	–78	Decomposition
2	*t*-BuLi, HMPA	THF	–78	Decomposition
3	*t*-BuLi, DMPU	THF	–78	Decomposition
4	*i*-PrMgCl.LiCl	THF	0	Decomposition
5	AIBN, (*n*-Bu_3_)SnH	Toluene	50	70%
6	BEt_3_, (*n*-Bu_3_)SnH	Toluene	–50	90%

**Table 2:** Screened reaction conditions for the Fries rearrangement of **139**

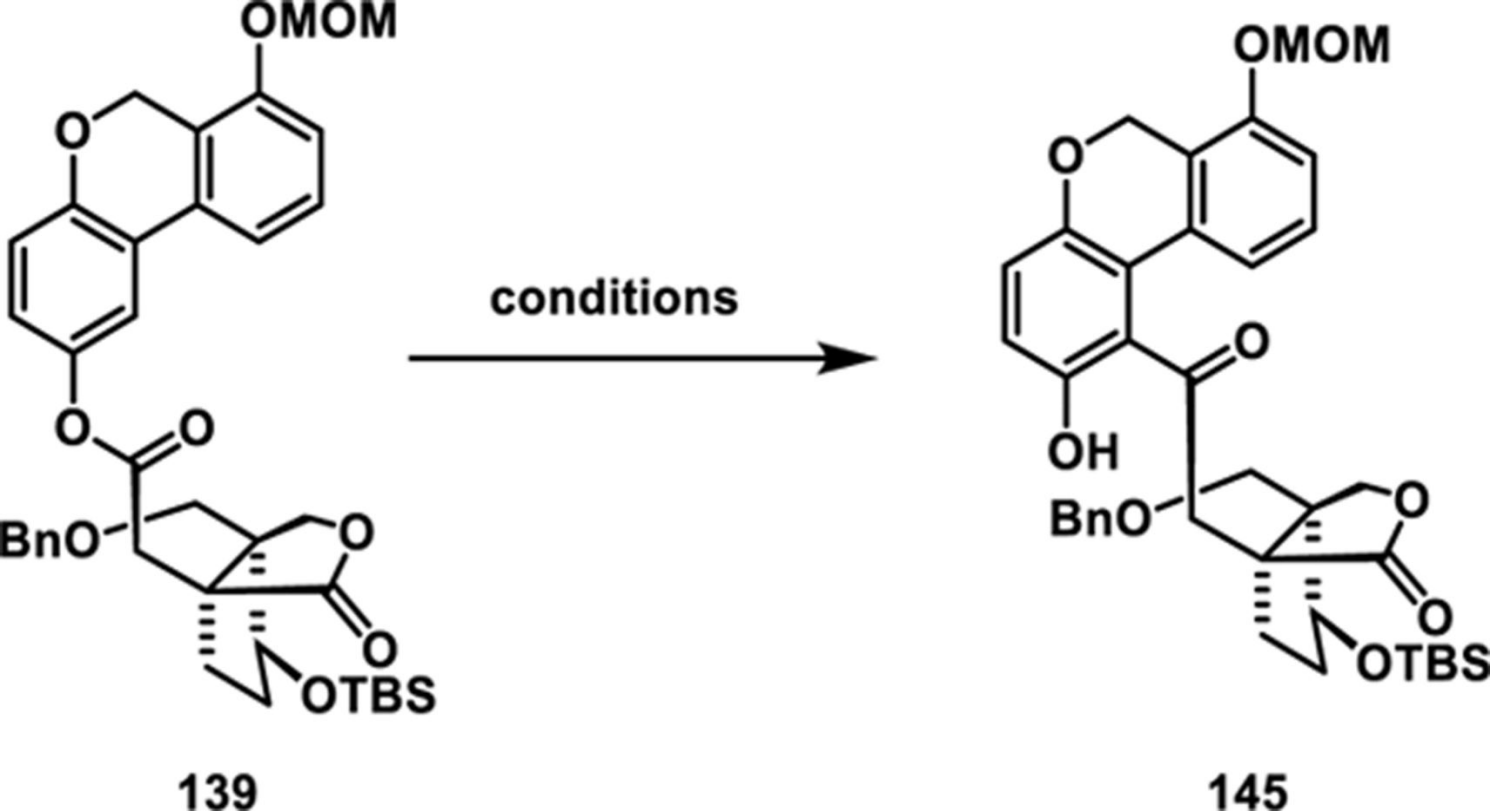				
Entry	Conditions	Solvent	*T* [°C]	Observation
1	Sc(OTf)_3_	1,2-dichlorobenzene	150	Decomposition
2	AlCl_3_	1,2-dichlorobenzene	150	Decomposition
3	BF**_3_**.OEt**_2_**	1,2-dichlorobenzene	150	Decomposition
4	254 nm	MeCN	23	Traces of **145**
5	254 nm	Cyclohexane	23	Traces of **145**
6	254 nm	MeOH	23	8%

## Data Availability

The data that support the findings of this study are available in the supplementary material of this article.

## Footnotes

Supporting information for this article is available on the WWW under https://doi.org/10.1002/ceur.202500020
